# Integration of machine learning to develop a disulfidptosis model for predicting glioma prognosis, immunotherapy response, and drug

**DOI:** 10.1016/j.isci.2026.115657

**Published:** 2026-04-08

**Authors:** Ruiting Huang, Hailin Li, Yijing Zhong, Paimin Zhuo, Yibei Wang, Aoting Yang, Yu Zhang, Jiao Li, Ruiquan Xu, Quhuan Li

**Affiliations:** 1 School of Biology and Biological Engineering, South China University of Technology, Guangzhou, Guangdong 510006, China; 2 The First Clinical School of Gannan Medical University, Ganzhou, Jiangxi 341000, China; 3 Gannan Medical University, Ganzhou, Jiangxi province 341000, China; 4 Department of Urology, First Affiliated Hospital of Gannan Medical University, Ganzhou, Jiangxi 341000, China; 5 Longnan First People's Hospital, Longnan, Jiangxi 341700, China

**Keywords:** bioinformatics, cancer, artificial intelligence applications

## Abstract

Glioma prognosis is challenged by tumor heterogeneity and lack of biomarkers. Disulfidptosis, a novel cell death mechanism induced by disulfide stress, remains poorly understood in gliomas. This study analyzed eight glioma cohorts, identifying two disulfidptosis patterns with distinct genomic alterations, immune microenvironments, and clinical outcomes. A prognostic model—DisulfidpScore—was developed using machine learning, demonstrating robust predictive ability for survival. Crucially, single-cell profiling and virtual knockout analysis revealed elevated disulfidptosis in glioblastoma astrocytes and identified *IQGAP1* as a key driver that modulates gene networks governing the cell cycle and neuron-glia interactions. High DisulfidpScore scores correlated with immunosuppressive microenvironments and poorer prognosis but increased chemotherapy sensitivity, whereas low scores indicated better survival and immunotherapy response. The model supports prognostic stratification and personalized treatment for glioma patients.

## Highlights

• Two disulfidptosis patterns identified in gliomas
• Develops a prognostic model, DisulfidpScore, using 101 machine learning algorithms
• Links disulfidptosis to immunosuppressive microenvironment in gliomas
• A tool for personalized glioma prognosis and management

## Graphical abstract

**Figure:**
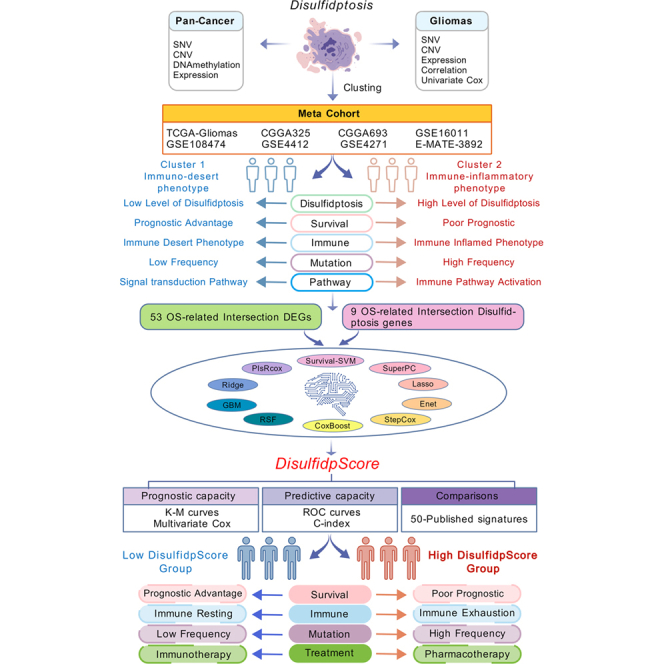

## Introduction

Gliomas represent one of the predominant primary malignant brain tumors within the central nervous system, accounting for nearly 80% of all malignant brain neoplasms, and they are a major focus in neuro-oncology.^1^ Gliomas are characterized by high heterogeneity, aggressive behavior, and malignancy, resulting in high recurrence and mortality rates. In 2021, the World Health Organization (WHO) classified gliomas into four grades (I to IV), with WHO grades II and III defined as low-grade gliomas (LGGs) and WHO grade IV designated as glioblastoma (GBM). Among these, GBM is the most malignant and has the poorest prognosis, with a 5-year survival rate of merely 5%.^2^ Furthermore, nearly 70% of patients with LGG experience disease progression within 10 years, leading to worse prognosis.^3^ The conventional treatment protocol for gliomas is surgical resection, followed by radiotherapy and chemotherapy. However, even with this multimodal approach, the median survival time for patients with GBM post-treatment remains at only 14.6 months.^4^ With ongoing research, immunotherapy has been successful in treating patients with gliomas. Nevertheless, the complex and highly immunosuppressive microenvironment of gliomas means that only a minority of patients derive significant benefit from immunotherapy.^5^ Although immune checkpoints, tumor mutational burden (TMB), microsatellite instability (MSI), neoantigen load (NAL), and mismatch repair deficiency (DMMR) have the potential to predict the efficacy of immune checkpoint inhibitors (ICIs), their application is restricted due to spatiotemporal heterogeneity, moderate accuracy, and limited patient populations.^6^ Additionally, gliomas exhibit treatment resistance due to epigenetic and tumor microenvironmental heterogeneity.^7^ Consequently, it is essential to identify novel therapeutic targets and establish individualized assessment systems for precise treatment of gliomas.

Disulfidptosis is a recently identified mode of cell death that primarily occurs under conditions of cellular metabolic dysregulation or abnormal redox environments. Disulfidptosis is distinct from other known forms of cell death in that it is not influenced by conventional cell death inhibitors.^8^ Liu et al. documented that cancer cells with increased *SLC7A11* protein experience insufficiency in NADPH when subjected to glucose deprivation.^9^ Consequently, these cells are unable to effectively process excess cystine, leading to intracellular disulfide stress. Disulfide stress is characterized by the abnormal accumulation of disulfide bonds in actin cytoskeletal proteins, resulting in the collapse of the actin cytoskeleton and ultimately triggering disulfidptosis.^9^ The discovery of disulfidptosis offers new perspectives for understanding disease mechanisms and developing therapeutic strategies. Recent research has highlighted the significant link between disulfidptosis and a range of diseases, such as cancers, cardiovascular conditions, neurodegenerative diseases, and liver pathologies.^10^ In the context of cancer, disulfidptosis is closely associated with patient treatment response and prognosis.^11^ Although existing studies have unveiled the therapeutic potential of disulfidptosis in cancer treatment, the mechanisms underlying disulfidptosis and its possible therapeutic uses in gliomas still need to be explored further.

This study was designed to explore the correlation between disulfidptosis and the occurrence and progression of gliomas, as well as to develop a prognostic model that would enable patients to receive more accurate treatment. Initially, we identified 15 genes related to disulfidptosis via a comprehensive literature review. Subsequently, we employed the GSCA (Gene Set Cancer Analysis) database to investigate the transcriptional and genetic profiles of these genes across various cancers. In parallel, leveraging data from The Cancer Genome Atlas (TCGA), we examined the mutational landscapes, differential expression patterns, intergene correlations, and prognostic potential of disulfidptosis-related genes specifically within gliomas. Subsequently, we utilized unsupervised clustering analysis to discern distinct disulfidptosis patterns in gliomas and scrutinized the variations in these patterns concerning the expression levels of disulfidptosis-related genes, survival prognosis, immune infiltration, gene mutations, and signaling pathways. Furthermore, we integrated machine learning algorithms to construct and identify the best disulfidptosis-related prognostic model, DisulfidpScore, and evaluated its prognostic and predictive performance. Additionally, we compared this model with previously published glioma-related models to assess its superiority. Finally, we explored the model’s changes in immune infiltration and genomic features, and we assessed its potential for predicting responses of patients with gliomas to immunotherapy and their sensitivity to chemotherapy. Importantly, to elucidate cellular heterogeneity and specific regulatory mechanisms at single-cell resolution, we performed single-cell RNA sequencing (scRNA-seq) analysis and conducted virtual knockout experiments to investigate the gene regulatory networks driven by key disulfidptosis regulators.

## Results

### Transcriptional and genetic characteristics of 15 disulfidptosis genes

**Figure 1:**
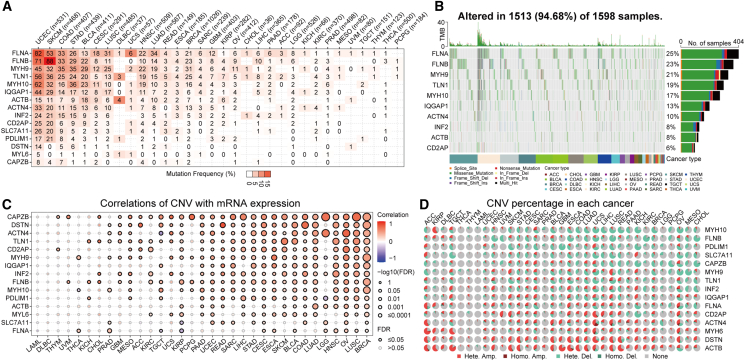
Transcriptomic and epigenetic landscape of disulfidptosis genes in pan-cancer data (A) Mutation frequency of disulfidptosis genes in pan-cancer data. Numbers in the figure indicate the number of harmful mutations, with blank spaces indicating no mutations in all regions of the gene. (B) Mutation frequency, types, and distribution of the top ten disulfidptosis genes with the highest mutation frequency in pan-cancer data. (C) Association between disulfidptosis gene expression and their CNVs in pan-cancer data. (D) Distribution of CNV types of disulfidptosis genes in pan-cancer. HETE Amp, heterozygous amplification; HETE Del, heterozygous deletion; Homo Amp, homozygous amplification; Homo Del, homozygous deletion; None, no CNV.

First, we examined the transcriptional and genetic characteristics of disulfidptosis genes across pan-cancer data. Regarding SNVs, the highest frequency of harmful mutations in disulfidptosis genes was observed in Uterine Corpus Endometrial Carcinoma (UCEC), Skin Cutaneous Melanoma (SKCM), and Colon Adenocarcinoma (COAD), whereas the mutation frequency in LGGs and GBMs was less than 3% (Figure 1A). The waterfall plot indicates the top ten disulfidptosis genes with the highest mutation frequency across cancers, with *FLNA* exhibiting the highest mutation frequency (25%), followed by *FLNB* (23%) and *MYH9* (21%). Missense mutations were the most common mutation type (Figure 1B). A significant positive correlation was observed between the copy number variations (CNVs) of disulfidptosis genes and their expression levels in most cancers, including LGGs and GBMs (Figure 1C). Additionally, disulfidptosis genes exhibited various degrees of heterozygous or homozygous amplification or deletion across all cancers (Figure 1D), with heterozygous amplification or deletion being the primary CNV type (Figures S1A and S1B). Almost all cancers exhibited a significant negative correlation between disulfidptosis gene expression and DNA methylation levels (Figure S1C). Expression differences revealed that disulfidptosis genes possessed significant expression variations in most cancers, with *SLC7A11* exhibiting significant upregulation in most cancers (Figure S1D). These findings suggest a significant link between the transcriptional imbalance of disulfidptosis-related genes and genetic diversity across various cancer types.

**Figure 2:**
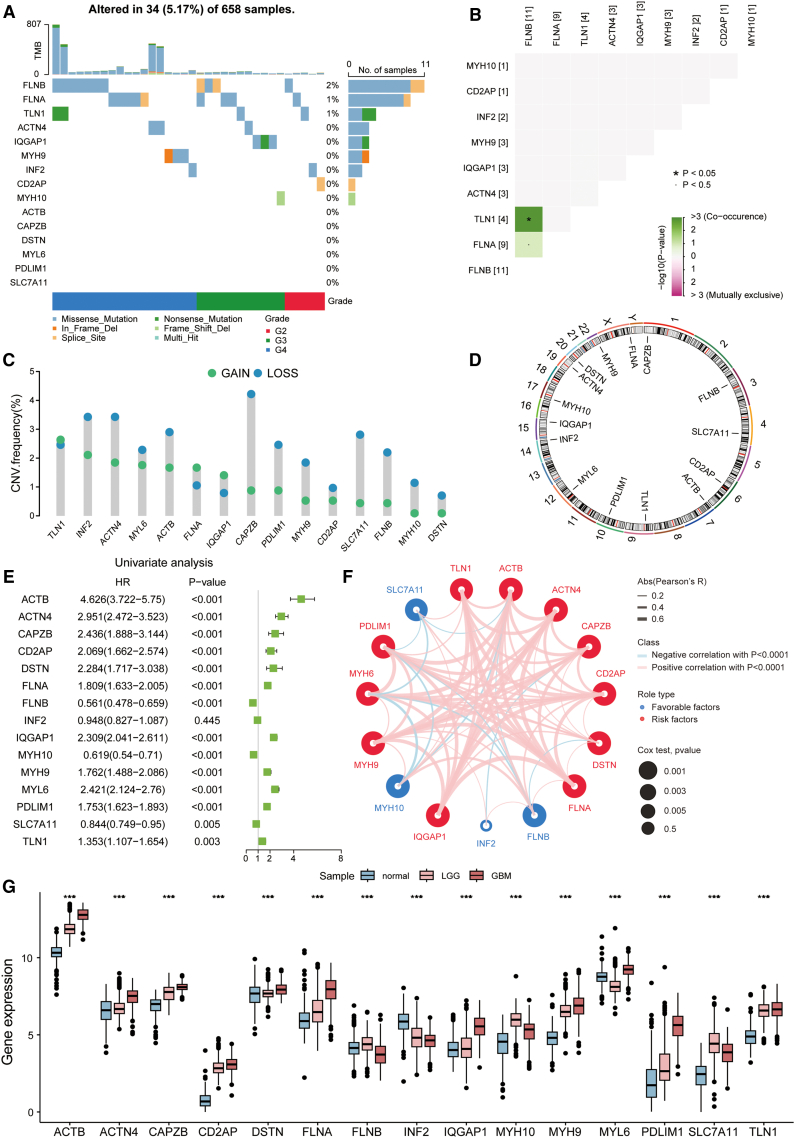
Multidimensional characteristics of disulfidptosis genes in gliomas (A) Mutation frequency and types of disulfidptosis genes in gliomas. (B) Mutation correlations among disulfidptosis genes in gliomas using Fisher’s exact test, with green indicating co-mutation and purple indicating mutually exclusive mutation. (C) CNV frequency and types of disulfidptosis genes in gliomas. (D) Chromosomal distribution of disulfidptosis genes. (E) Univariate Cox regression analysis results of disulfidptosis genes in gliomas. (F) Interaction among disulfidptosis genes in gliomas. The thickness of the lines indicates the strength of the correlation, where pink lines denote positive correlations and blue lines signify negative correlations. Red and blue circles indicate risk factors and protective factors, respectively, with circle size representing prognostic value. (G) Expression differences of disulfidptosis genes in normal brain, LGG, and GBM samples analyzed using Wilcoxon rank-sum test. ∗∗∗, *p* < 0.001.

Next, we focused on the effect of disulfidptosis genes on gliomas. Among 658 patients with glioma, only 34 (5.17%) possessed SNVs, with *FLNB* exhibiting the highest mutation frequency of just 2%. This was followed by *FLNA* (1%) and *TLN1* (1%), whereas no mutations were observed in the remaining genes. Missense mutations were the predominant SNV type, and SNVs were more frequent in patients with grade 4 gliomas (Figure 2A). Apart from the co-mutation relationship between *TLN1* and *FLNB*, no significant mutation relationships were observed among other disulfidptosis genes (Figure 2B). CNVs were present in all disulfidptosis genes, with most exhibiting a higher frequency of deletions than amplifications, except for *TLN1*, *FLNA*, and *IQGAP1* that exhibited opposite trends (Figure 2C). Chromosomal circle plots revealed that the CNVs of these genes primarily occurred on chromosome 1, 3, 4, 6, 7, 9, 10, 12, 14, 15, 17, 19, 20, and 22 (Figure 2D). Univariate Cox regression analysis indicated that all disulfidptosis genes, except for *INF2*, had significant prognostic value in gliomas. Specifically, *FLNB*, *MYH10*, and *SLC7A11* were protective factors for glioma prognosis, while the remaining 11 disulfidptosis genes were risk factors (Figure 2E). Correlation analysis revealed significant positive correlations among most disulfidptosis genes (Figure 2F). Additionally, glioma samples and normal brain samples showed markedly different expression of disulfidptosis genes. Compared to normal samples, *ACTB*, *ACTN4*, *CAPZB*, *CD2AP*, *DSTN*, *FLNA*, *IQGAP1*, *MYH10*, *MYH9*, *PDLIM1*, *SLC7A11*, and *TLN1* were upregulated in gliomas, *FLNB* was upregulated in LGGs but downregulated in GBMs, *MYH6* was downregulated in LGGs but upregulated in GBMs, and *INF2* was downregulated in gliomas (Figure 2G; STAR Methods). Furthermore, the expression of disulfidptosis genes was investigated with respect to glioma disease grade. The results demonstrated that *ACTB*, *ACTN4*, *CAPZB*, *CD2AP*, *FLNA*, *DSTN*, *IQGAP1*, *MYH9*, *MYH6*, *PDLIM1*, and *TLN1* were significantly upregulated with increased disease severity, whereas *FLNB*, *INF2*, and *SLC7A11* exhibited significant downregulation. *MYH10* was initially upregulated and then downregulated with disease severity (Figures S2A–S2O). These findings suggest that disulfidptosis genes are closely related to glioma patient prognosis and that CNVs may be a major cause of transcriptional dysregulation of disulfidptosis genes.

### Unsupervised clustering to identify different disulfidptosis patterns in gliomas

**Figure 3:**
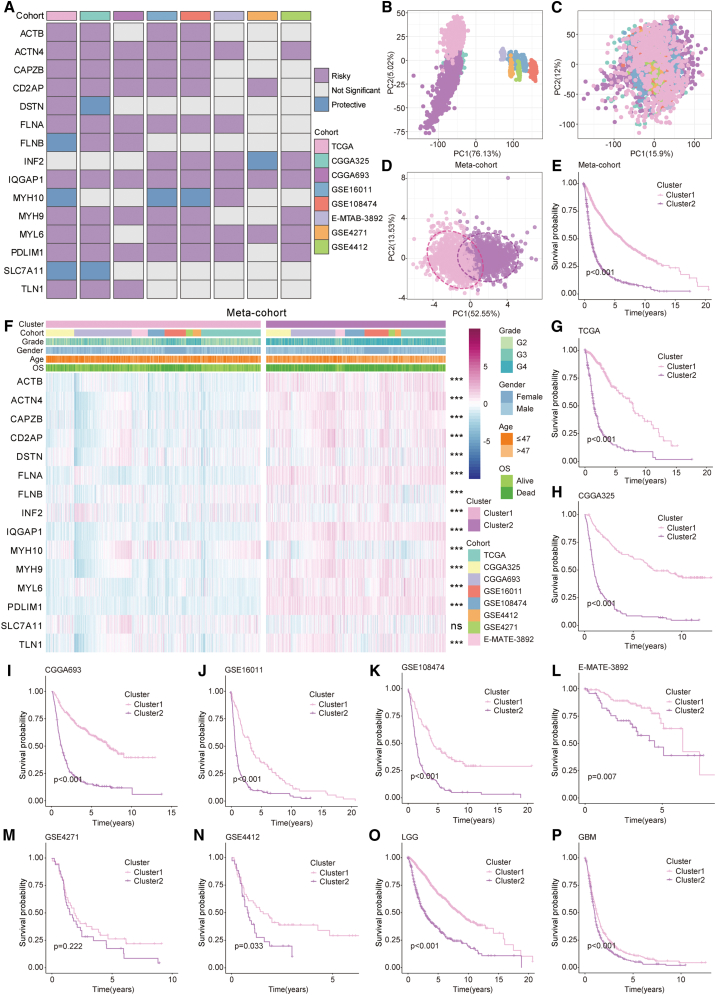
Classification characteristics and survival prognosis of different disulfidptosis patterns in gliomas (A) Prognostic value of disulfidptosis genes in eight glioma cohorts. Purple and blue labels indicate risk factors and protective factors, respectively, with gray labels indicating no prognostic value. (B and C) Sample distribution in the meta-cohort before and after removing batch effects. (D and E) Sample distribution differences and survival differences between two disulfidptosis patterns in the meta-cohort. (F) Expression levels of disulfidptosis genes and distribution of clinical characteristics under two different disulfidptosis patterns analyzed using Wilcoxon rank-sum test. (G–P) Survival differences between two disulfidptosis patterns in TCGA, CGGA325, CGGA693, GSE16011, GSE108474, E-MATE-3892, GSE4271, GSE4412, LGG, and GBM cohorts, analyzed using the Kaplan-Meier method with log-rank test. ∗∗∗, *p* < 0.001; ns, not statistically significant.

We performed univariate Cox regression analysis on 15 disulfidptosis genes across eight GBM cohorts in order to obtain a thorough understanding of the regulatory role of these genes in gliomas and to investigate an emerging categorization of glioma patients based on these genes. We identified nine prognostic genes with *p* < 0.05 in five or more cohorts, including *ACTN4*, *CAPZB*, *CD2AP*, *FLNA*, *INF2*, *IQGAP1*, *MYH9*, *MYL6*, and *PDLIM1*, for clustering glioma patients (Figure 3A). Furthermore, we merged the eight glioma cohorts into a meta-cohort and used a combat algorithm to remove batch effects between the different cohorts. The principal-component analysis (PCA) results revealed the sample distribution before (Figure 3B) and after (Figure 3C) the removal of batch effects, indicating that the batch effects were effectively corrected. Given the potential biological differences between LGGs and GBMs, we extracted the LGG and GBM cohorts separately from the meta-cohort for further analysis and validation.

Based on the expression levels of the nine prognostic disulfidptosis genes, *k*-means unsupervised clustering analysis identified two distinct disulfidptosis patterns in the meta-cohort, labeled cluster 1 and cluster 2. The PCA results revealed substantial differences between samples in the two patterns (Figure 3D) that were validated across the eight cohorts as well as in the LGG and GBM cohorts (Figures S3A–S3J). We also compared the molecular expression and clinicopathological parameters of different disulfidptosis patterns to explore their potential associations. The results indicated that most disulfidptosis genes had significantly higher expression levels in cluster 2 than in cluster 1, with the exceptions of *MYH10* that expressed at low levels in cluster 2 and *SLC7A11* that exhibited no difference in expression between the two groups (Figure 3F; STAR Methods). We observed that patients in cluster 2 generally exhibited higher disease grades, older age, and more frequent death events that were also reflected in the eight independent cohorts, as well as in the LGG and GBM cohorts (Figure S3K). To visually assess the impact of different disulfidptosis patterns on patient prognosis, we conducted a survival analysis.

The results indicated that patients in cluster 2 had a significantly worse prognosis (Figure 3E), and cluster 2 showed a trend of poor prognosis in the other nine cohorts (Figures 3G–3P; STAR Methods). However, no association between disulfidptosis patterns and survival differences was observed in the GSE4271 cohort, which might be explained by the limited sample size of this cohort. Therefore, our cluster analysis based on nine prognostic disulfidptosis genes identified two distinct patterns in gliomas. Cluster 2 was associated with higher disulfidptosis gene expression, poorer prognosis, and more severe clinical manifestations.

### Immune infiltration differences in gliomas with different disulfidptosis patterns

To evaluate the role of different disulfidptosis patterns in the glioma immune microenvironment and explore potential immune mechanisms, we conducted a systematic analysis. We found that cluster 2 had a greater level of immune cell infiltration based on six different immune infiltration analysis algorithms (Figure S4A). The ESTIMATE results indicated that both the immune and stromal scores were significantly higher in cluster 2 than in cluster 1 (Figure S4B). An essential mechanism for the immune system to identify and eliminate tumor cells is the cancer immunity cycle, which includes steps such as tumor antigen release, tumor antigen presentation, T cell priming and activation, T cell transport to tumors, T cell infiltration into tumors and stroma, T cell recognition of target tumor cells, and T cell killing of tumor cells. These steps must be coordinated for effective tumor control and elimination. All seven steps were consistently upregulated in cluster 2 (Figure S4C). In terms of immune function, cluster 2 exhibited significantly higher immune functional activity than cluster 1 (Figure S4D). Next, we looked into how different disulfidptosis patterns differed in terms of immune cell infiltration. The results revealed that M2 macrophages, regulatory T (Treg) cells, CD8^+^ T cells, resting memory CD4^+^ T cells, and resting NK cells exhibited higher infiltration abundances in cluster 2, whereas monocytes and activated NK cells exhibited more significant infiltration in cluster 1 (Figure S4E). In tumor immune microenvironment (TIME) feature analysis, cluster 2 exhibited higher levels of glycolysis, IFN-γ response, myeloid-derived suppressor cells (MDSCs), Treg cells, innate immunity, tumor recognition and proliferation (as developed by Kobayashi) (Figure S4F), angiogenesis, antigen presentation, cancer-associated fibroblasts (CAFs), immune checkpoints, granulocytes, tumor-promoting immune infiltration, and tumor characteristics (as developed by Bagaev) (Figure S4G). These findings uncover the immune microenvironment characteristics associated with different disulfidptosis patterns in gliomas, suggesting that cluster 2 may be more prone to an immunoinflammatory phenotype, whereas cluster 1 is more akin to an immune-desert phenotype. These findings are important for understanding tumor immune evasion mechanisms and developing effective immunotherapy strategies.

### Genomic characteristic differences in gliomas with different disulfidptosis patterns

We explored the differences in genomic characteristics between different disulfidptosis patterns in gliomas. We observed that the frequencies of both CNV amplifications and deletions were higher in cluster 2 than in cluster 1 (Figures S5A and S5B). Regarding specific chromosomal changes, cluster 1 exhibited a higher frequency of 1p19q co-deletion (Figure S5C) that is a typical genomic marker of oligodendrogliomas, whereas cluster 2 exhibited higher frequencies of chr7 gain and loss of chr10 (Figure S5D) and characteristic genomic alterations of GBMs, consistent with the disease grade distribution among the different disulfidptosis patterns. Overall, cluster 2 possessed a significantly higher CNV frequency than cluster 1 (Figure S5E). In terms of SNVs, cluster 2 exhibited a higher TMB than cluster 1 (Figure S5F). These differences may pose significant implications for tumor biological behavior and the selection of treatment strategies.

### Signaling pathway differences in gliomas with different disulfidptosis patterns

To uncover the potential differential mechanisms and functional pathways of different disulfidptosis patterns in gliomas, we first conducted gene set variation analysis (GSVA) based on hallmark gene sets. The results revealed that the pathways associated with tumor occurrence and metastasis were significantly activated in cluster 2, including the epithelial-mesenchymal transition and angiogenesis pathways. Moreover, immune- and inflammation-related pathways, including inflammatory response, IL-6/JAK/STAT3 signaling, interferon α/γ (IFN-α/γ) signaling, TNF-α signaling, and allograft rejection, were also abnormally active in cluster 2. Cell cycle-related pathways such as E2F targets and the G2M checkpoint were also activated in cluster 2. In contrast, cluster 1 was associated with the negative regulation of the KRAS signaling pathway (Figure S6A).

Next, we contrasted how the two patterns differed in their oncogenic signaling pathways. In cluster 2, TP53, TGF-β, PI3K, NRF2, Notch, Hippo, and cell cycle pathways were significantly enriched, while Wnt, RAS, and MYC pathways were more prominent in cluster 1 (Figure S6B). To precisely identify the specific pathways of the different disulfidptosis patterns, we performed gene set enrichment analysis (GSEA). The results showed that cluster 1 was markedly enriched in the neural signaling pathways, including calcium signaling, gap junctions, long-term potentiation, and neuroactive ligand-receptor interactions (Figure S6C). Cluster 2 was primarily associated with immune pathways, including chemokine signaling, cytokine-cytokine receptor interaction, ECM-receptor interaction, adhesion molecules, Toll-like receptor signaling, hematopoietic cell lineage, and JAK-STAT signaling (Figure S6D). These results provide a distinct perspective of the biological characteristics of different glioma disulfidptosis patterns.

### Model construction based on the disulfidptosis patterns

To further investigate disulfidptosis-related genes, we conducted a differential analysis between the different disulfidptosis patterns in the eight glioma cohorts. We identified 295 differentially expressed genes that were upregulated or downregulated in seven or more cohorts and defined them as glioma-specific disulfidptosis-related genes.

**Figure 4:**
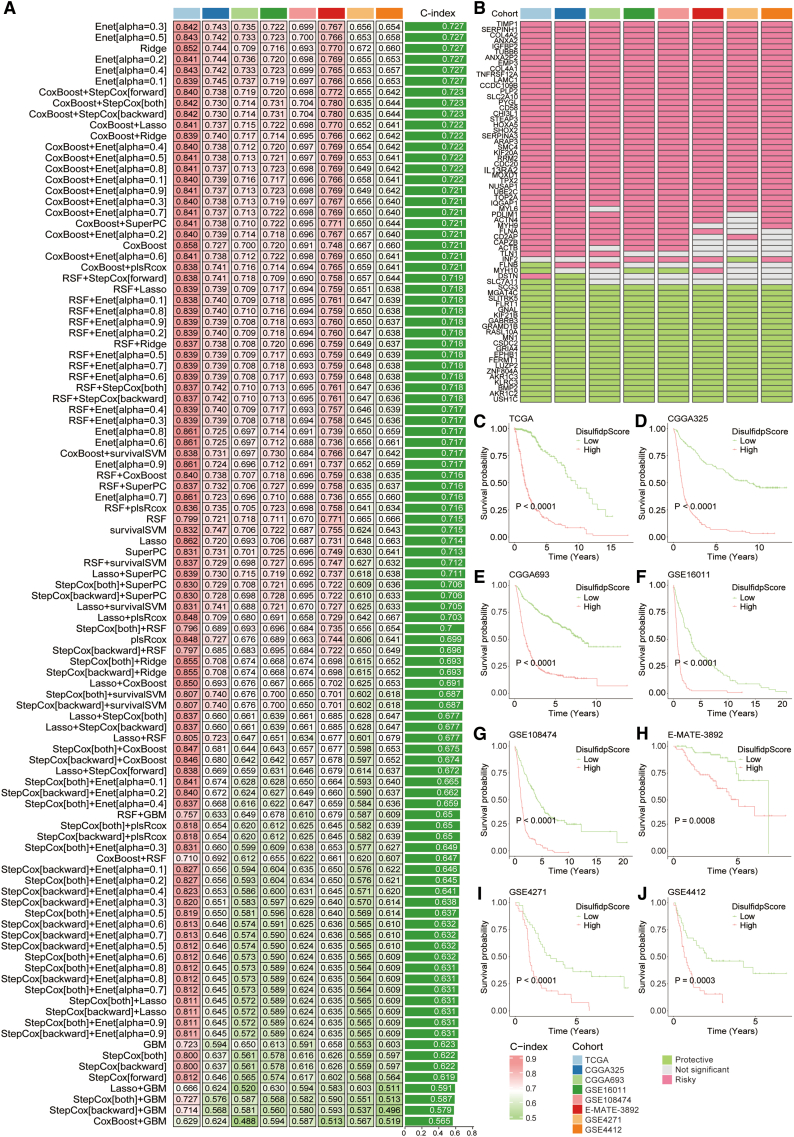
Model construction and prognostic evaluation of DisulfidpScore in gliomas (A) C-index of 101 algorithm combination models established by integrating ten machine learning algorithms in eight glioma cohorts, with the average C-index on the right column. (B) Prognostic value of 53 prognostic differential genes and nine prognostic disulfidptosis genes in eight cohorts with different disulfidptosis patterns in gliomas. (C–J) Survival differences between different DisulfidpScore score groups in TCGA, CGGA325, CGGA693, GSE16011, GSE108474, E-MATE-3892, GSE4271, and GSE4412 cohorts, analyzed using the Kaplan-Meier method with log-rank test.

Subsequently, univariate Cox regression analysis identified 53 common prognostic disulfidptosis-related genes across the eight cohorts (Figure 4B). These 53 prognostic differential genes and nine prognostic disulfidptosis genes were then used to develop a disulfidptosis-related model, DisulfidpScore, using a machine learning-based approach. Specifically, we integrated ten machine learning algorithms, including RSF, Enet, StepCox, CoxBoost, PlsRcox, SuperPC, GBM, Survival-SVM, Ridge, and LASSO, resulting in 101 algorithm combinations for model construction and selection. Across all datasets, each candidate model was rigorously evaluated via 10-fold cross-validation to prevent overfitting and ensure internal validity. Each model in the TCGA training set and seven validation sets (CGGA325, CGGA693, GSE16011, GSE108474, GSE4271, GSE4412, and E-MATE-3892) had its C-index determined. To determine the DisulfidpScore score, we chose Enet[alpha = 0.3] with the greatest average C-index (Figure 4A).

### Prognostic ability of DisulfidpScore

Glioma patients were divided into high- and low-DisulfidpScore score groups based on the median DisulfidpScore score in order to evaluate the prognostic performance of DisulfidpScore. Overall survival rates were considerably worse for patients in the high-DisulfidpScore score group than for those in the low-DisulfidpScore score group across the seven validation sets and the training set, according to survival analysis (Figures 4C–4J; STAR Methods). Multivariate Cox regression analysis, which incorporated the DisulfidpScore score and clinical characteristics of gliomas, indicated that the DisulfidpScore score is an independent prognostic factor for gliomas, with an HR greater than 1, making it an independent risk factor. However, the results in the E-MATE-3892, GSE4271, and GSE4412cohorts were not statistically significant due to the limited sample sizes (Figures S7A, S7C, S7E, S7G, S7I, S7K, S7M, and S7O). Additionally, we explored the distribution differences of clinical characteristics in the different DisulfidpScore score groups, including age, sex, disease grade, IDH mutation status, 1p19q co-deletion status, and MGMTp (O-6-methylguanine-DNA methyltransferase promoter) methylation status. According to the analysis, patients in the high-DisulfidpScore score group had considerably higher disease grades, older age, and more frequent IDH mutations, 1p19q co-deletions, and MGMTp methylation, whereas age distribution did not significantly differ between the DisulfidpScore score groups (Figures S7B, S7D, S7F, S7H, S7J, S7L, S7N, and S7P). DisulfidpScore has good prognostic performance according to these results.

### Predictive ability of DisulfidpScore

**Figure 5:**
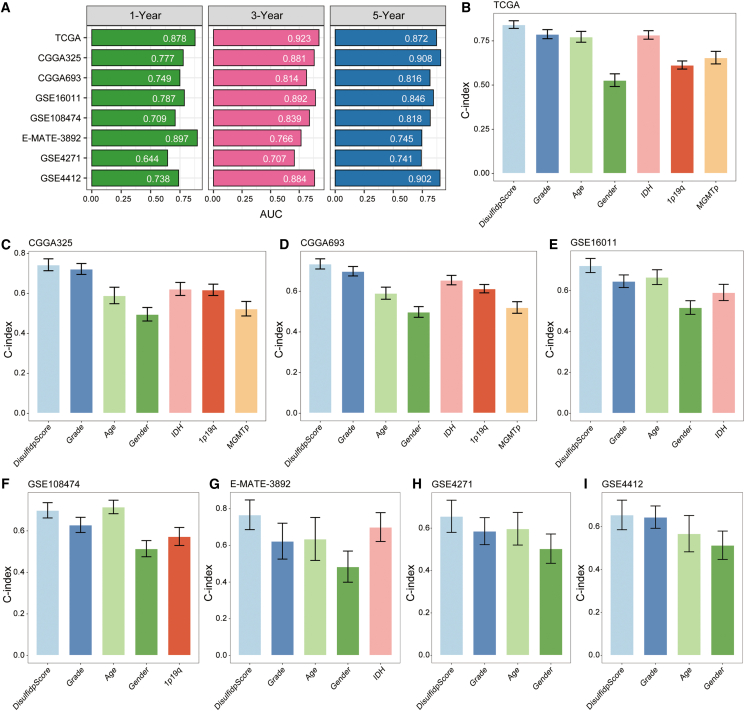
Predictive ability evaluation of DisulfidpScore in gliomas (A) AUC values of eight glioma cohorts of 1-, 3-, and 5-year ROC curves for DisulfidpScore scores. (B–I) C-index of DisulfidpScore score compared with those of clinical and molecular characteristics of gliomas in TCGA, CGGA325, CGGA693, GSE16011, GSE108474, E-MATE-3892, GSE4271, and GSE4412 cohorts.

We plotted time-dependent ROC curves for the DisulfidpScore score in the eight glioma cohorts to assess the predictive performance of DisulfidpScore. Specifically, the areas under the curve (AUC) for the 1-, 3-, and 5-year ROC curves were 0.878, 0.923, and 0.872, respectively, in the TCGA. Similarly, the AUC values were 0.777, 0.881, and 0.908 in the CGGA325 cohort; 0.749, 0.814, and 0.816 in the CGGA693 cohort; 0.787, 0.892, and 0.846 in the GSE16011 cohort; 0.709, 0.839, and 0.818 in the GSE108474 cohort; 0.897, 0.766, and 0.745 in the E-MATE-3892 cohort; 0.644, 0.707, and 0.741 in the GSE4271 cohort; and 0.738, 0.884, and 0.902 in the GSE4412 cohort (Figure 5A). AUC values exceeding 0.7 were considered to indicate good predictive accuracy of DisulfidpScore. In clinical practice, certain clinical and molecular characteristics are often utilized for prognostic evaluations, clinical staging, and treatment decision-making.^12^ Together, these results indicated that DisulfidpScore exhibits robust and consistent prognostic performance across multiple independent cohorts. Notably, the 3- and 5-year AUC values exceeded or approached 0.8 in most cohorts, with particularly high discrimination in several datasets. Although the 1-year AUC was relatively low in some cohorts, the overall trend supports the reliability of DisulfidpScore in stratifying patient outcomes over time and across different glioma populations. Therefore, we further compared the performance of DisulfidpScore with the clinical and molecular characteristics of gliomas in predicting prognosis. The results demonstrated that the C-index of the DisulfidpScore score was higher than that of the glioma clinical and molecular characteristics in all eight cohorts (Figures 5B–5I). Thus, we conclude that DisulfidpScore demonstrates reliable prognostic predictive performance in patients with glioma.

### Comparison of DisulfidpScore with other glioma-related models

**Figure 6:**
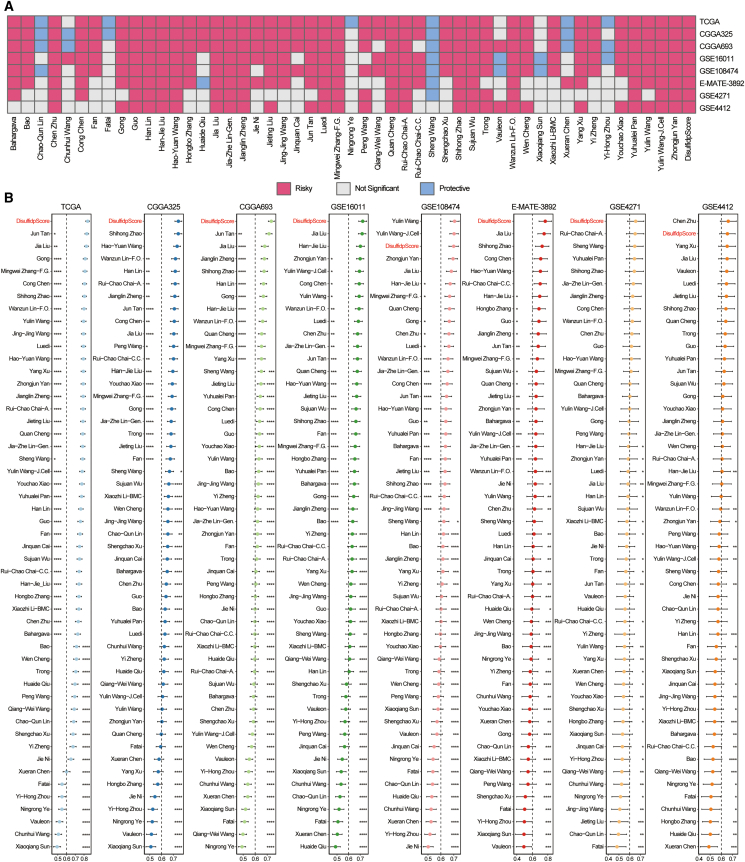
Comparison of DisulfidpScore with 50 other glioma signatures (A) Prognostic value of DisulfidpScore and 50 other glioma signatures across eight cohorts. (B) C-index of DisulfidpScore and 50 other glioma signatures across eight cohorts, analyzed using paired *t* test or Wilcoxon signed-rank test. ∗∗∗∗, *p* < 0.0001; ∗∗∗, *p* < 0.001; ∗∗, *p* < 0.01; ∗, *p* < 0.05.

To comprehensively evaluate the performance differences between DisulfidpScore and other glioma-related models, we collected 50 glioma-related models published over the past decade (Figure 6). These models involve various biological processes, including RNA-binding proteins, hypoxia, IFNs, autophagy, glycolysis, immune infiltration, synapse-related proteins, ferroptosis, ion channels, the extracellular matrix, N6-methyladenosine (m6A), 5-methylcytosine (m5C), glioma stem cells, transcription factors, polyadenylation, glucose metabolism, and immune checkpoints. Through univariate Cox regression analysis, we observed that 19 models, including DisulfidpScore, possessed significant prognostic value across the eight cohorts (Figure 6A). Further comparison revealed that DisulfidpScore demonstrated superior predictive accuracy in almost all cohorts. It achieved the highest C-index in TCGA, CGGA325, CGGA693, GSE16011, E-MATE-3892, and GSE4271 cohorts and ranked second in GSE4412 and third in GSE108474 (Figure 6B; STAR Methods). It is noteworthy that although most of the models performed well on the training set and some validation sets, their performance was suboptimal in some validation sets, likely owing to overfitting that led to insufficient model generalization. Overall, these results indicated that the DisulfidpScore could reliably and precisely predict the prognosis of glioma patients and possesses certain advantages over other models, providing a powerful emerging tool for the clinical management of glioma patients.

### Immune infiltration characteristics associated with DisulfidpScore scores in gliomas

We investigated potential relation between the immunological microenvironment and DisulfidpScore scores. Because of the extensive clinical information and multiomics data provided by TCGA cohort, we selected it as the primary study subject. We also conducted parallel analyses in the meta-cohort and the LGG and GBM cohorts to increase the comprehensiveness of the analysis.

**Figure 7:**
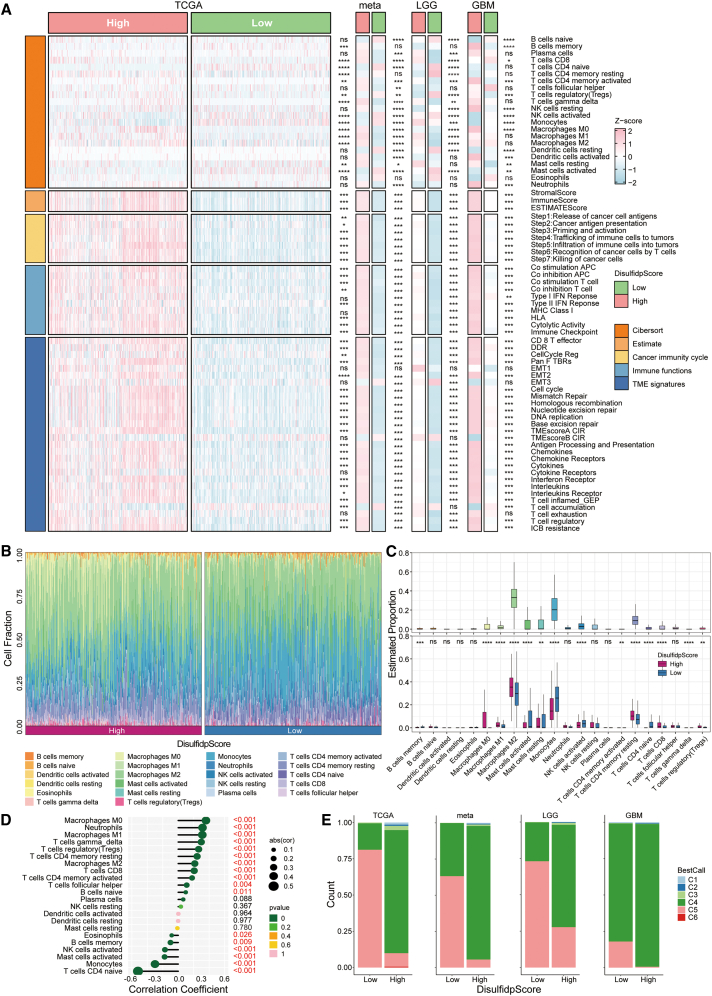
Immune infiltration changes associated with DisulfidpScore score in gliomas (A) Differences in immune infiltration among different DisulfidpScore score groups analyzed using Wilcoxon rank-sum test or *t* test, including immune cell infiltration, ESTIMATE scores, cancer immunity cycle, immune function, and TIME features. (B) Distribution of immune cell infiltration proportions among different DisulfidpScore score groups. (C) Differences in immune cell infiltration between different DisulfidpScore score groups, analyzed using Wilcoxon rank-sum test. (D) Correlation between DisulfidpScore score and immune cells. Circle size indicates the strength of correlation, and circle color corresponds to the *p* value. (E) Distribution of immune subtypes among different DisulfidpScore score groups in TCGA, meta (eight independent glioma cohorts), LGG, and GBM cohorts, using chi-square test or Fisher’s exact test. ∗∗∗∗, *p* < 0.0001; ∗∗∗, *p* < 0.001; ∗∗, *p* < 0.01; ∗, *p* < 0.05; ns, not statistically significant. C1, wound healing; C2, IFN-γ dominant; C3, inflammatory; C4, lymphocyte depletion; C5, immunologically quiet; C6, TGF-β dominant.

First, we focused on the characteristics of the immune microenvironment based on DisulfidpScore scores. In the high-DisulfidpScore score group, we observed richer immune cell infiltration. In the ESTIMATE analysis, the group with high DisulfidpScore scores also had higher stromal, immunological, and ESTIMATE scores. Additionally, the high-DisulfidpScore score group showed enhanced cancer immunity cycle activity and upregulated immune functions. Further TIME analysis showed that the group with a high DisulfidpScore score had higher amounts of epithelial-mesenchymal transition, tumor microenvironment scores, and immune regulators (including chemokines, cytokines, and IFNs). Notably, the high-DisulfidpScore score group had higher T cell exhaustion scores, while the low-DisulfidpScore score group exhibited more significant T cell aggregation. The high-DisulfidpScore score group also had a higher immune checkpoint blockade (ICB) resistance score, indicating a poor response to ICI treatment. Similar findings were observed in the meta-cohort, LGG cohort, and GBM cohort analyses (Figure 7A).

Subsequently, we used CIBERSORT software to quantify the infiltration abundance of 22 immune cell types. The results revealed that macrophages, monocytes, and T cells were the predominant types of immune cell infiltrates, with M2 macrophages and resting CD4^+^ memory T cells exhibiting the highest proportions. The high-DisulfidpScore score group exhibited significantly higher infiltration levels of M2 macrophages and Treg cells, suggesting enhanced immune evasion. Additionally, this group had significant enrichment of CD8^+^ T cells, M0 and M1 macrophages, and resting mast cells. In contrast, the low-DisulfidpScore score group exhibited higher infiltration levels of monocytes, activated mast cells, and activated NK cells, and this partly explained the better prognosis of this group (Figures 7B and 7C; STAR Methods). The results of the correlation analysis indicated that the DisulfidpScore score had a significant negative correlation with naive CD4^+^ T cells, monocytes, activated mast cells, activated NK cells, memory B cells, and eosinophils, and a significant positive correlation with M0, M1, and M2 macrophages, neutrophils, γδ T cells, Treg cells, resting CD4^+^ memory T cells, CD8^+^ T cells, activated CD4^+^ memory T cells, follicular helper T cells, and naive B cells (Figure 7D).

Tumor specimens can be classified into six different immune subtypes, including C1 (wound healing), C2 (IFN-γ dominant), C3 (inflammatory), C4 (lymphocyte depletion), C5 (immunologically quiet), and C6 (TGF-β dominant).^13^ Therefore, we identified the immune subtypes in patients with gliomas. The findings indicated that the predominant subtypes among glioma patients were C4 and C5. The C4 subtype was more prevalent in the high-DisulfidpScore score group, while the C5 subtype was more prevalent in the low-DisulfidpScore score group (Figure 7E; STAR Methods). In summary, the presence of many immunosuppressive cells may dominate the tumor immune evasion behavior, affecting the effectiveness of immunotherapy and patient prognosis, even though the high-DisulfidpScore score group had higher immune infiltration.

### Genomic alterations associated with DisulfidpScore scores in gliomas

**Figure 8:**
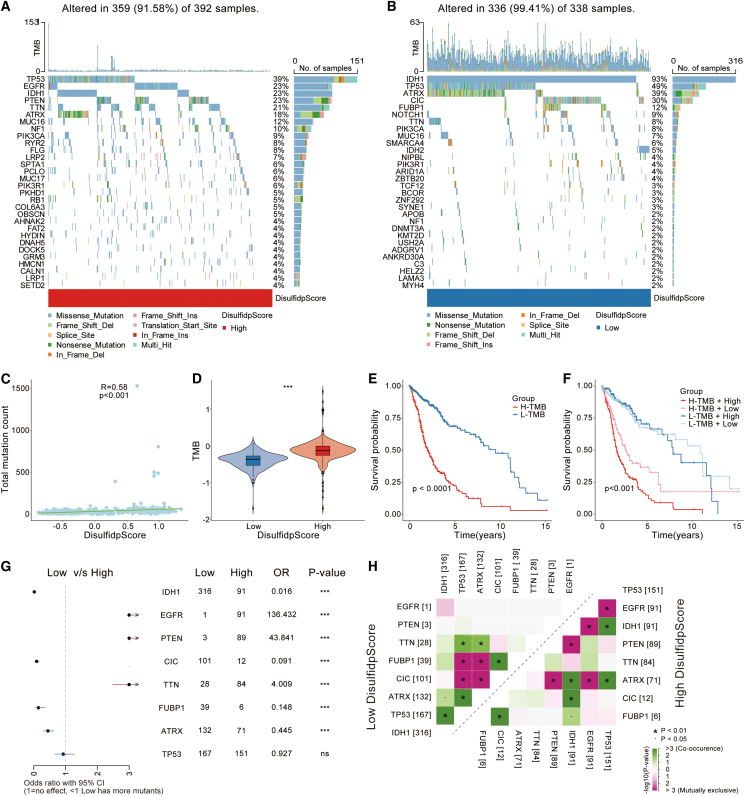
Genomic variation features associated with DisulfidpScore score in gliomas (A and B) Mutation frequency and types of the top 30 genes in (A) high-DisulfidpScore score and (B) low-DisulfidpScore score groups. (C) Correlation between DisulfidpScore scores and the total number of mutations, using Wilcoxon rank-sum test. (D) Differences in TMB between different DisulfidpScore score groups. (E) Survival differences between different TMB groups, analyzed using Kaplan-Meier method with log-rank test. (F) Survival differences between TMB groups combined with DisulfidpScore score grouping, analyzed using Kaplan-Meier method with log-rank test. (G) Mutation differences of key mutated genes, analyzed using Fisher’s exact test. (H) Mutation associations of key mutated genes between different DisulfidpScore score groups, analyzed using Fisher’s exact test. ∗∗∗, *p* < 0.001; ns, not statistically significant.

We investigated the genomic alterations associated with DisulfidpScore scores. Waterfall plots displayed the top 30 genes with the highest mutation frequencies and mutation types in both high- and low-DisulfidpScore score groups. In the high-DisulfidpScore score group, mutation frequencies were higher for genes such as *TP53*, *EGFR*, *PTEN*, and *TTN* that are associated with poor prognosis. Conversely, the low-DisulfidpScore score group exhibited higher mutation rates for genes such as *IDH1*, *ATRX*, *CIC*, and *FUBP1*, indicating better prognosis (Figures 8A and 8B). A strong positive correlation between the DisulfidpScore score and the total number of mutations in glioma patients was found by correlation analysis (Figure 8C; STAR Methods). Additionally, the TMB was calculated, and it was found that the group with a high DisulfidpScore score had a significantly higher TMB than the group with a low DisulfidpScore score (Figure 8D). Survival analysis further demonstrated that patients with high TMB experienced a poorer prognosis (Figure 8E; STAR Methods). Survival curves combining the DisulfidpScore score and TMB groups indicated that patients in the low-TMB and -DisulfidpScore score groups exhibited the best survival advantage, whereas those in the high-TMB and -DisulfidpScore score groups exhibited a significantly poorer prognosis (Figure 8F; STAR Methods).

Comparing the key mutated genes in the different DisulfidpScore score groups, we determined that mutations in *IDH1*, *CIC*, *FUBP1*, and *ATRX* were more prevalent in the low-DisulfidpScore score group, whereas *EGFR*, *PTEN*, and *TTN* had higher mutation rates in the high-DisulfidpScore score group (Figure 8G; STAR Methods). We also analyzed the co-occurrence and mutual exclusivity of key mutated genes in the different DisulfidpScore score groups that exhibited distinct patterns in the high- and low-DisulfidpScore score groups (Figure 8H; STAR Methods). These results suggested that the DisulfidpScore score is not only closely related to genomic alterations in gliomas but is also significantly associated with TMB and patient prognosis.

### Predictive ability of DisulfidpScore in glioma immunotherapy response

We assessed the predictive capacity of DisulfidpScore for immunotherapy response. As mentioned previously, the high-DisulfidpScore score group exhibited higher genomic alteration frequencies, TMB, and an active immune microenvironment, suggesting the potential efficacy of immunotherapy in this group. However, the high-DisulfidpScore score group also possessed a higher abundance of immunosuppressive cells, higher ICB resistance scores, and a higher frequency of *PTEN* mutations, and this could negatively affect the effectiveness of immunotherapy. On the basis of this, we hypothesized that patients with low DisulfidpScore scores could respond better to immunotherapy.

**Figure 9:**
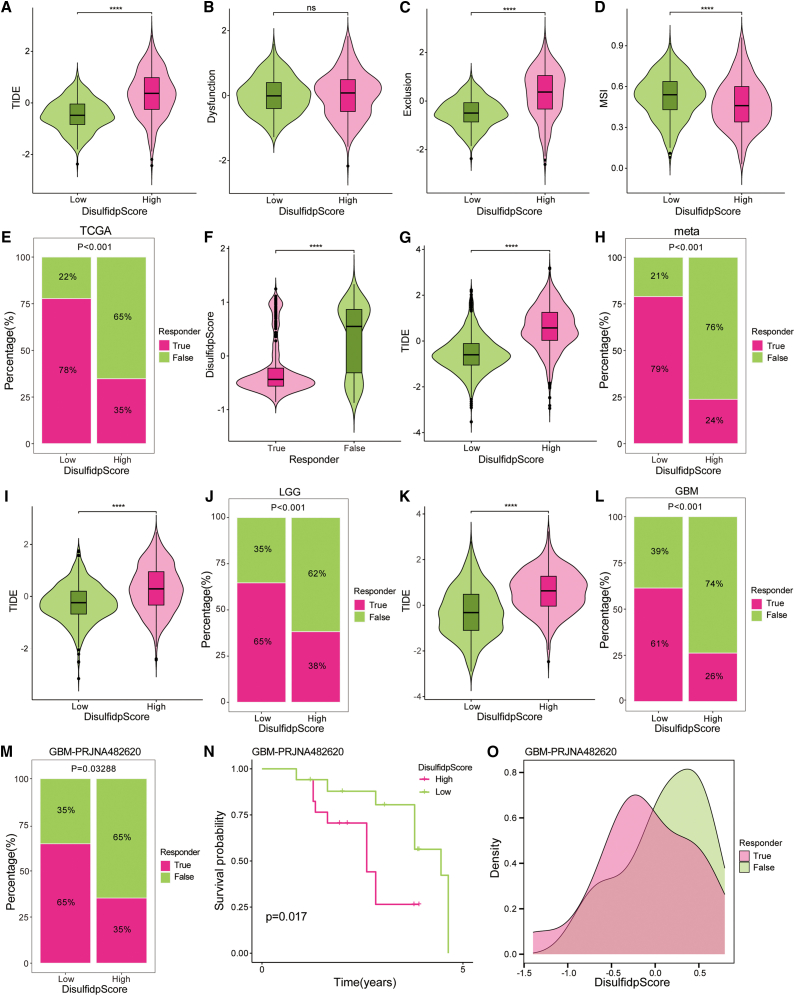
Predictive ability of DisulfidpScore for immunotherapy response in gliomas (A–D) TIDE, MSI, Exclusion, and Dysfunction scores among different DisulfidpScore score groups in the TCGA cohort, analyzed using Wilcoxon rank-sum test. (E) The proportion of patients with (pink) or without (green) immunotherapy response between different DisulfidpScore score groups in the TCGA cohort, analyzed using chi-square test. (F) DisulfidpScore score differences between patients with (pink) or without (green) immunotherapy response, analyzed using Wilcoxon rank-sum test. (G–L) Differences in TIDE and the percentage of patients who responded to immunotherapy or not among different DisulfidpScore score groups in the meta cohort (G and H), LGG cohort (I and J), and GBM cohort (K and L), analyzed using Wilcoxon rank-sum test and chi-square test, respectively. (M and N) The proportion of patients with (pink) or without (green) immunotherapy response and survival differences between different DisulfidpScore score groups in the GBM-PRJNA482620 cohort, analyzed using the chi-square test and Kaplan-Meier method with log-rank test, respectively. (O) DisulfidpScore score differences between patients with or without immunotherapy response in the GBM-PRJNA482620 cohort, analyzed using Wilcoxon rank-sum test. ∗∗∗∗, *p* < 0.0001; ns, not statistically significant.

We performed a TIDE analysis, in which lower TIDE scores suggest a reduced likelihood of immune escape and a greater chance of benefiting from immunotherapy. High MSI scores are associated with good response to immunotherapy. The results showed that the group with low DisulfidpScore scores had considerably lower TIDE scores than the group with high DisulfidpScore scores (Figure 9A; STAR Methods). There were not significant differences in the T cell dysfunction scores between the DisulfidpScore score groups (Figure 9B; STAR Methods); however, the high-DisulfidpScore score group had significantly higher T cell exclusion scores (Figure 9C; STAR Methods). Additionally, the low-DisulfidpScore score group exhibited higher MSI scores (Figure 9D; STAR Methods). The percentage stacked plot indicated that a higher proportion of patients in the low-DisulfidpScore score group responded to immunotherapy (Figure 9E; STAR Methods). We further compared the DisulfidpScore scores between responders and non-responders and determined that responders possessed significantly lower DisulfidpScore scores (Figure 9F; STAR Methods). Similar results were obtained in the meta-, LGG-, and GBM cohort analyses (Figures 9G–9L; STAR Methods).

Particularly in the GBM cohort treated with anti-PD-L1 therapy, the group with low DisulfidpScore scores had a larger percentage of patients receiving anti-PD-L1 treatment (Figure 9M; STAR Methods) and a better prognosis (Figure 9N; STAR Methods). Mechanistically, high-DisulfidpScore score tumors exhibited upregulation of key immune checkpoint genes (*PD-1*, *PD-L1*, *CTLA-4*, and *LAG-3*), whose elevated expression correlates with poor prognosis (Figures S8A and S8B) and positively correlates with the score itself (Figure S8C), further confirming its association with an immunosuppressive microenvironment. Further analysis revealed enhanced CD8^+^ T cell exhaustion in tumors with high DisulfidpScore scores, concurrent with increased effector T cell infiltration and heightened inhibitory Treg activity (Figures S8D and S8F). Collectively, these findings suggest that despite elevated TMB, the coexistence of T cell dysfunction and immunosuppression in the high-DisulfidpScore score cohort likely drives resistance to ICIs. Although the DisulfidpScore score was lower in the responder group, DisulfidpScore values were not significantly different between responders and non-responders (Figure 9O; STAR Methods). This may be attributed to its relatively limited sample size. These findings emphasize the potential significance of DisulfidpScore in predicting the response of glioma patients to immunotherapy, with those in the low-DisulfidpScore score group more likely to benefit.

### Deciphering disulfidptosis and *IQGAP1*-driven networks in gliomas at single-cell resolution

**Figure 10:**
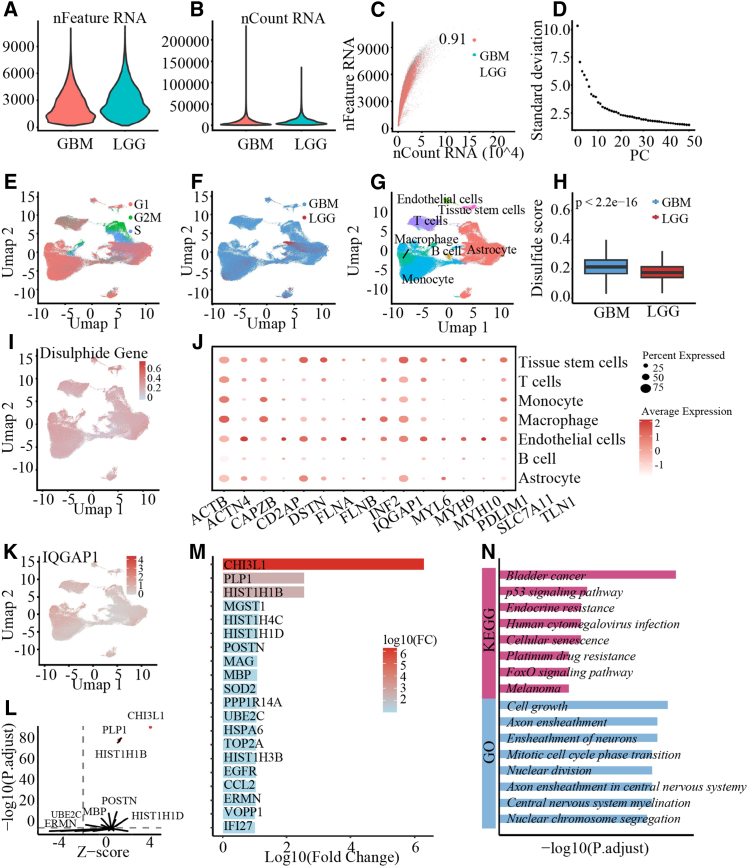
Single-cell profiling of disulfidptosis features and virtual knockout analysis of *IQGAP1* (A–D) Quality control and preprocessing metrics. Violin plots display the number of genes (A) and UMI counts (B) per cell, with their correlation shown in the scatterplot (C). Elbow plot indicates the optimal principal components for clustering (D). (E–G) Global characterization of the single-cell landscape. UMAP projections illustrate the distribution of cell cycle phases (E), sample groups (GBM vs. LGG) (F), and the annotated cell type clusters (G). (H–J) Evaluation of disulfidptosis signatures. The comparison of DisulfidpScore scores reveals higher levels in GBM than in LGG, analyzed using Wilcoxon rank-sum test (H), while the expression patterns of disulfidptosis-related genes are visualized via feature plot (I) and a dot plot (J) across different cell types. (K–N) Virtual knockout (KO) analysis of *IQGAP1*. (K) The baseline expression of *IQGAP1* shown on the UMAP plot. (L) The genome-wide perturbation scatterplot. (M) The top 20 genes with the most significant regulatory shifts. (N) The associated functional enrichment pathways following *IQGAP1* KO.

To elucidate the cellular heterogeneity and transcriptional dynamics of disulfidptosis in gliomas, we performed scRNA-seq analysis on the GBM and LGG samples. After rigorous quality control and filtration based on gene counts and mitochondrial content (Figures 10A–10D), we obtained a high-quality single-cell transcriptome atlas. Dimensionality reduction via UMAP visualized the distinct distribution of cell cycle phases (Figure 10E) and sample groups (Figure 10F). Cell type annotation using SingleR identified seven major clusters: astrocytes, B cells, endothelial cells, macrophages, monocytes, T cells, and tissue stem cells (Figure 10G). We next evaluated the activity of the disulfidptosis gene signature, using the AUCell algorithm. Notably, the DisulfidpScore score was significantly upregulated in GBM compared with LGG (Figure 10H), suggesting a positive correlation between disulfidptosis levels and tumor malignancy. Feature plots and dot plots further highlighted the distinct expression patterns of core disulfidptosis genes across these cell subpopulations (Figures 10I and 10J).

Given that *IQGAP1* was identified as a consistent high-risk factor across all eight datasets in our previous analysis, we focused on its regulatory role specifically in astrocytes. We performed *in silico* knockout analysis, using scTenifoldKnk, to interrogate its function (Figure 10K). The virtual depletion of *IQGAP1* induced widespread perturbations in the gene regulatory network (Figure 10L). Specifically, the top 20 genes with the most significant regulatory shifts included *CHI3L1*, *PLP1*, and *HIST1H1B* (Figure 10M). Functional enrichment analysis revealed that these perturbed genes were primarily enriched in biological processes related to cell growth and division (e.g., mitotic cell cycle phase transition, nuclear division, and nuclear chromosome segregation), neural development (e.g., axon ensheathment and CNS myelination), and cancer-related signaling (e.g., p53 signaling pathway, FoxO signaling pathway, and cellular senescence) (Figure 10N). These findings suggested that *IQGAP1* is essential for maintaining the proliferative potential and malignant phenotype of glioma cells.

### Predictive ability of DisulfidpScore for chemotherapy drug sensitivity in gliomas

**Figure 11:**
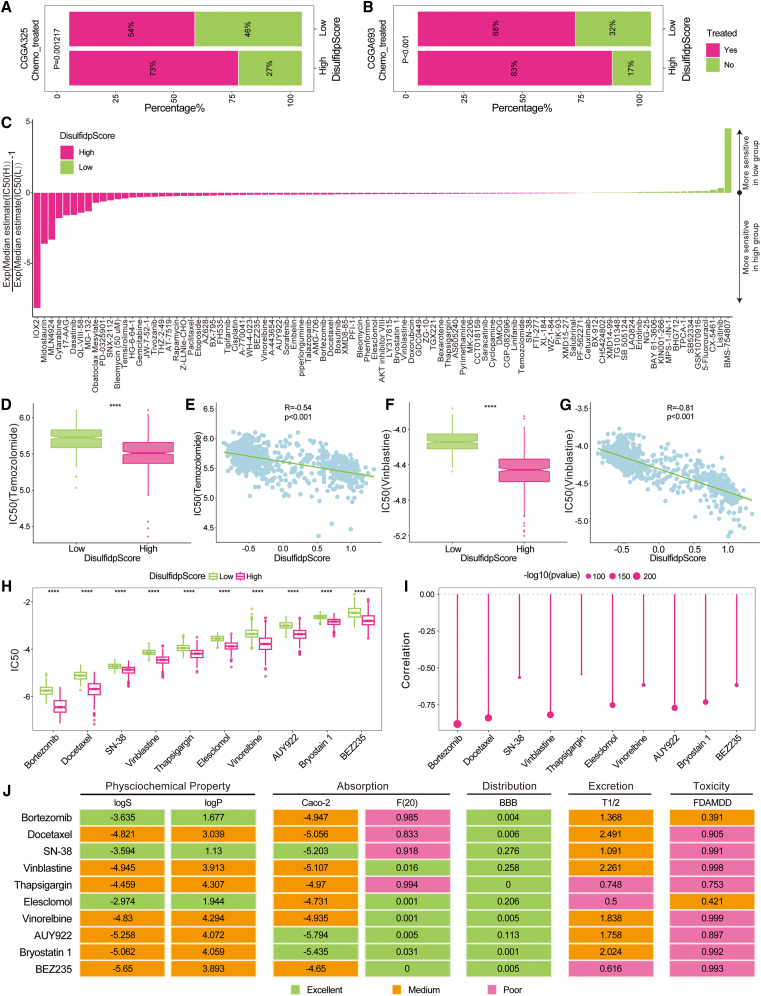
Predictive ability of DisulfidpScore for chemotherapy drug sensitivity in gliomas (A and B) The proportion of patients who received chemotherapy for different DisulfidpScore score groups in the CGGA325 and CGGA693 cohorts, analyzed using chi-square test. (C) Normalized IC_50_ values of 95 drugs. (D) IC_50_ values of temozolomide in different DisulfidpScore score groups, analyzed using Wilcoxon rank-sum test. (E) Correlation between DisulfidpScore scores and temozolomide IC50 values. (F) IC_50_ values of vincristine in different DisulfidpScore score groups, analyzed using Wilcoxon rank-sum test. (G) Correlation between DisulfidpScore scores and vincristine IC_50_ values. (H) IC_50_ values of 10 candidate therapeutic drugs in different DisulfidpScore score groups, analyzed using Wilcoxon rank-sum test. (I) Correlation between DisulfidpScore scores and IC_50_ values of 10 candidate therapeutic drugs. (J) ADMET properties of 10 candidate therapeutic drugs. ∗∗∗∗, *p* < 0.0001.

We evaluated the predictive ability of DisulfidpScore for the sensitivity of glioma patients to chemotherapy drugs. Given that the clinical data from the CGGA cohort may include information on patients who had received chemotherapy, we compared the proportion of patients receiving chemotherapy in the different DisulfidpScore score groups. The results showed that a noticeably greater proportion of patients in the high-DisulfidpScore score group received chemotherapy, in both the CGGA325 and CGGA693 cohorts (Figures 11A and 11B; STAR Methods). Furthermore, we assessed the potential therapeutic benefit of 251 drugs for gliomas, using the pRRophetic package, and identified 95 drugs with significantly different IC50 values between the different DisulfidpScore score groups. We found that the group with high DisulfidpScore scores tended to be sensitive to more drugs (Figure 11C). Temozolomide and vincristine are first-line treatment drugs for gliomas.^14^^,^^15^ We observed that the IC50 values for these two drugs were lower in the high-DisulfidpScore score group and extremely negatively correlated with the DisulfidpScore score, implying that patients in the group with high DisulfidpScore scores would respond better to these drugs (Figures 11D–11G; STAR Methods).

Considering the heterogeneity in drug sensitivity between the different DisulfidpScore score groups, we further explored potential drugs that exhibited higher sensitivity in the high-DisulfidpScore score group, defining the ten drugs with the lowest IC50 values as candidate drugs. The analysis indicated that the group with high disulfidptosis scores exhibited more sensitivity to these ten candidate drugs (Figure 11H; STAR Methods), with their IC50 values showing a significant negative correlation with the DisulfidpScore score (Figure 11I). Notably, the inclusion of vincristine as an established first-line treatment further validates the reliability of our results. Subsequently, we comprehensively evaluated the physicochemical properties and pharmacokinetic characteristics of candidate drugs. The analysis indicated that in addition to vincristine, the most promising drug was elesclomol, which was followed by AUY922 and boratezamib (Figure 11J). To further evaluate the drug-like properties and translational potential of these candidates, we performed a comprehensive ADMET (absorption, distribution, metabolism, excretion, toxicity) analysis. As summarized in Figure 11J, elesclomol displayed favorable pharmacokinetic characteristics, including balanced predictions for oral bioavailability, blood-brain barrier penetration, and low cytotoxicity risk. Although AUY922 and bortezomib showed slightly elevated values in certain toxicity or metabolic metrics, their overall profiles remain within acceptable ranges, suggesting potential for further development. Importantly, the inclusion of the clinically established agent vincristine among the sensitive drugs reinforces the biological relevance of our prediction model. Collectively, these findings demonstrated that DisulfidpScore not only stratifies glioma patients into subgroups with distinct drug-response profiles but also assists in prioritizing pharmacokinetically promising compounds for future experimental evaluations. This supports the potential utility of DisulfidpScore in predicting chemotherapy drug sensitivity in glioma patients and offers valuable insights for personalized treatment strategies.

## Discussion

The prognosis of gliomas remains poor owing to their high heterogeneity that leads to differences in aggressive progression and treatment responses.^7^ Therefore, there is an urgent need to identify new targets or develop new predictive models to establish individualized assessment systems. These systems can help predict patient prognosis, guide targeted therapy, and study resistance mechanisms, thereby enabling the formulation of personalized treatment strategies. The findings on disulfidptosis provide a distinct perspective on cancer metabolic treatment. By evaluating the conditions of disulfidptosis in tumor patients and establishing disulfidptosis-related models, it is possible to provide guidance for the prognostic evaluation of diseases and innovate cancer treatment strategies.

We explored the transcriptomic and genetic characteristics of disulfidptosis genes at the pan-cancer level and observed significant differences in most cancers (Figure S1). According to available data, disulfidptosis can influence the occurrence and progression of tumors,^16^^,^^17^^,^^18^^,^^19^ and it is closely associated with non-cancer diseases.^20^^,^^21^ Current research has shown that the activity of disulfidptosis varies across different tumor types. In gliomas, the expression of several key genes—including *GYS1*, *RPN1*, *LRPPRC*, *NFE2L3*, *LINC00641*, and *LYRM4-AS1*—is closely linked to patient prognosis and cellular functions. Among these, *LINC00641* is downregulated in glioma and suppresses tumor growth through the *miR-4262*-*NRGN* pathway. Moreover, *GYS1* has been found to regulate tumor cell proliferation, migration, and oxidative stress, indicating its potential role in disulfidptosis through modulation of the metabolic, apoptotic, and inflammatory processes. These findings underscore *GYS1* and other related genes as promising molecular targets for glioma therapy.^22^ However, glioma is characterized by high biological heterogeneity, and the mechanism by which these genes collectively shape the tumor landscape remains to be elucidated.

Moving beyond individual gene analysis, this study employed unsupervised clustering analysis to reveal two distinct disulfidptosis patterns in gliomas that exhibited significant differences in the immune microenvironment, genomic variations, and signaling pathways (Figures S4–S6). Compared with cluster 1, cluster 2 possessed higher expression levels of disulfidptosis genes, poorer prognosis, a higher frequency of gene mutations, and an immune-inflammatory phenotype in the immune microenvironment, with corresponding abnormally activated immune signaling pathways. Notably, in the evaluation of tumor prognosis and immunotherapy response, an active immune microenvironment and high TMB predict a better prognosis.^23^ The contradictory results in the immune microenvironment may be related to immune evasion behaviors in cluster 2 due to highly infiltrated immunosuppressive cells such as M2 macrophages, Treg cells, CAFs, and MDSCs. In the brain tumor microenvironment, tumor-associated macrophages (TAMs) are the predominant immune cell group,^24^ and studies have demonstrated that inhibiting TAMs can effectively block the progression of gliomas.^25^ At the genomic level, cluster 2 exhibited a higher frequency of CNVs and TMB. Although high TMB is typically linked to a good response to immunotherapy,^23^ in cluster 2, high TMB did not translate into a survival advantage. Our study found that the frequency of CNVs was significantly higher in cluster 2 than in cluster 1 and that the expression of most disulfidptosis-related genes (e.g., *SLC7A11* and *IQGAP1*) was positively correlated with their CNV status (Figures 2C and 2G) and associated with chromosomal instability (Figure 2D). Previous studies have shown that CNVs may directly alter gene dosage effects or indirectly affect the transcriptional regulatory networks of neighboring genes by inducing chromosomal instability, leading to dysregulated expression of key metabolic and redox-related genes such as *SLC7A11*.^26^ Therefore, we propose that CNVs may serve as an important upstream event driving the dysregulation of disulfidptosis-related gene expression, thereby affecting intracellular disulfide homeostasis and contributing to the more aggressive phenotype observed in cluster 2. Additionally, high CNV frequency may be associated with increased genetic instability of the tumor^27^ that could promote tumor aggressiveness and the development of resistance. At the signaling pathway level, disulfidptosis-related genes such as *SLC7A11* are not only involved in intracellular redox regulation but have also been reported to influence cytokine secretion and immune cell recruitment in the tumor microenvironment. Studies have shown that upregulated *SLC7A11* in GBMs leads to elevated extracellular glutamate levels, thereby promoting the proliferation, activation, and immunosuppressive function of Treg cells and enhancing intratumoral immunosuppression,^28^ which is consistent with our immune infiltration analysis in cluster 2 (Figure S4A). Thus, aberrant expression of disulfidptosis-related genes may actively shape an immunosuppressive microenvironment through metabolic-immune crosstalk, thereby weakening antitumor immune responses. Overall, these findings suggest that the anti-inflammatory tumor microenvironment and reduced antitumor effects in cluster 2 led to a poorer prognosis in glioma patients.

In current research, investigators often select specific modeling algorithms based on personal preferences. Although these models perform well, they often lack multicohort validation, and this may result in overfitting and cause certain limitations in clinical applications.^29^ To address this, we utilized 101 combinations of 10 machine learning algorithms, fitting models using one training set and seven validation sets, and we established the DisulfidpScore model by using the top-performing algorithm “Enet[alpha = 0.3],” using the highest average C-index. Furthermore, the prognostic capability of DisulfidpScore was demonstrated through survival and multivariate Cox regression analyses (Figure 4). Time-dependent ROC curves and comparisons with clinical features further confirmed superior predictive performance (Figure S7). Notably, we collected 50 published glioma-related models and compared their C-indices, indicating that DisulfidpScore consistently ranked among the top three across all cohorts, highlighting its superior and stable predictive performance (Figure 6). According to literature review, this study is the first attempt to integrate multiple machine learning algorithms to create a model of glioma, DisulfidpScore. IDH, 1p19q, and MGMTp status are considered biomarkers for prognostic evaluation and clinical strategies in patients with glioma.^30^^,^^31^ The DisulfidpScore score outperformed these known biomarkers in prognostic prediction and surpassed clinical features such as disease grade, age, and sex (Figure 5). This indicates that the optimal fit model’s feature genes and algorithm selection through multiple algorithm combinations ensure the predictive stability of the model. DisulfidpScore translates the emerging biology of disulfidptosis into an actionable clinical tool. It not only stratifies patient prognosis more effectively than current biomarkers but also pinpoints an immunotherapy-resistant subgroup likely to benefit from alternative treatments. Built on clinically accessible data, this model advances a precision-medicine workflow from risk prediction to therapeutic decision-making.

Given the limitations of most models in clinical applications, we conducted a thorough analysis of the clinical application potential of DisulfidpScore, particularly focusing on its predictive ability for immunotherapy response and drug sensitivity. There are two distinct mechanisms of immune escape in tumors. One involves the inhibition of T cell infiltration by immunosuppressive factors, and the other involves the presence of high levels of cytotoxic T cell infiltration into the tumor. However, these T cells are functionally inactive. TIDE predicts tumor immune escape by comprehensively assessing the activity of these two mechanisms.^32^ Lower TIDE scores indicated a reduced likelihood of immune escape and an increased probability of benefiting from immunotherapy. Our analysis demonstrated that the low-DisulfidpScore score group possessed lower TIDE scores, which suggested that they responded better to immunotherapy (Figure 9). Mechanistically, high-DisulfidpScore score tumors displayed upregulation of key immune checkpoint molecules, elevated T cell exhaustion, and enhanced immunosuppressive activity (Figure S8), which collectively contribute to immunotherapy resistance despite high immune infiltration. Analysis of the immunotherapy cohort GBM-PRJNA482620 provided additional evidence that the DisulfidpScore score is a reliable predictor of immunotherapy response. Although our DisulfidpScore demonstrates predictive potential for immunotherapy response, its validation in the current anti-PD-L1-treated GBM cohort was constrained by the cohort’s small sample size, which may have limited the statistical power to detect significant differences. Therefore, future validation in larger, prospective immunotherapy cohorts is essential to confirm and strengthen these findings.

To further unravel the cellular heterogeneity and molecular roots underlying these prognostic and therapeutic differences, we integrated scRNA-seq analysis. This analysis validated the elevated disulfidptosis activity in GBM compared with that in LGG, aligning perfectly with the immunosuppressive microenvironment (M2 macrophages and Tregs) and poor prognosis observed in the high-DisulfidpScore score bulk cohort. This suggests that malignant cells with high disulfidptosis burdens may actively recruit suppressor cells via the chemokine pathways identified in cluster 2. Crucially, *in silico* knockout of *IQGAP1* in astrocytes offers a specific mechanistic basis for the dysregulated pathways seen in bulk analysis. The collapse of mitotic and p53 signaling networks upon *IQGAP1* depletion mirrors the enrichment of cell cycle and oncogenic signatures (E2F, G2M, and TGF-β) in high-risk patients. Furthermore, perturbations in *CHI3L1* and *PLP1* (axon ensheathment) directly explain the activation of ECM-receptor interactions and the suppression of normal neural signaling observed in the bulk transcriptome, pinpointing *IQGAP1* as a pivotal hub linking disulfidptosis to tumor proliferation and invasion. Overall, our study demonstrates that DisulfidpScore can be used for risk stratification, prognostic evaluation, immunotherapy response prediction, and candidate drug sensitivity prediction in patients with gliomas, providing a powerful tool for personalized glioma treatment.

In terms of drug sensitivity prediction, we found that a greater percentage of patients in the high-DisulfidpScore score group received chemotherapy and exhibited higher sensitivity to multiple chemotherapy drugs (Figure 11). On the basis of this, we screened personalized candidate drugs for patients with a high DisulfidpScore score and evaluated their pharmacokinetic characteristics and *in vivo* safety. The comprehensive results indicated that elesclomol is the most promising therapeutic drug. Elesclomol is an anticancer drug that targets mitochondrial metabolism and induces oxidative stress. However, recent studies have suggested that it may exert anticancer effects by inducing cuproptosis.^33^^,^^34^ In gliomas, researchers have reported that combining elesclomol with TMZ significantly enhances cytotoxicity compared to TMZ alone^34^ and that elesclomol primarily inhibits glioma growth by inducing cuproptosis.^35^ Disulfidptosis and cuproptosis, while driven by distinct molecular triggers, are both tightly regulated by cellular redox homeostasis and metabolic stress. Through a literature review, we note that ferroptosis inducers synergize with copper ionophores to enhance cell death. Notably, elesclomol, a known inducer of cuproptosis that promotes lipoylated *DLAT* aggregation, not only drives cuproptosis but also amplifies cell death when combined with ferroptosis inducers. Furthermore, copper-induced proteotoxic stress contributes to both cuproptosis and the modulation of ferroptosis via *GPX4* degradation.^36^^,^^37^ Given that disulfidptosis similarly arises from severe oxidative and metabolic disruption, we hypothesize that the redox-perturbing capacity of elesclomol may extend to influencing disulfidptosis sensitivity, potentially by exacerbating shared stress pathways or depleting key metabolic cofactors. This interplay could position elesclomol as a dual-modulator capable of co-targeting disulfidptosis and cuproptosis in certain tumors, though this hypothesis warrants further mechanistic and functional validation.

Several avenues remain to extend this work. First, while our scRNA-seq analysis established elevated disulfidptosis activity in GBM astrocytes and identified *IQGAP1*-driven regulatory networks, the intercellular communication mechanisms through which these cells shape the immunosuppressive microenvironment warrant deeper investigation. Ligand-receptor interaction analysis (e.g., CellChat and NicheNet) would clarify signaling between disulfidptosis-active astrocytes and M2 macrophages or Tregs. Second, integration of spatial transcriptomics—when such data become available for gliomas—would map DisulfidpScore score distribution across tumor regions and clarify relationships with immune cell localization and therapeutic resistance. Third, focused analysis of promoter methylation at core disulfidptosis genes would complement our CNV findings and provide a more complete picture of transcriptional dysregulation. Fourth, development of a streamlined PCR-compatible gene signature (3–5 genes) and an interactive online calculator is underway to enhance clinical translation. Fifth, validation within molecularly defined subgroups (IDH, MGMT, and 1p19q) will establish the incremental value of DisulfidpScore beyond existing biomarkers. Finally, direct comparison with ferroptosis-, cuproptosis-, and necroptosis-based models, as well as preliminary pan-cancer application, will clarify the unique biological niche and generalizability of DisulfidpScore-based prognostic stratification.

### Limitations of the study

This study possesses certain limitations. First, it is important to note that all analyses are based on retrospective data from public databases. While DisulfidpScore was validated across multiple independent glioma cohorts, the retrospective nature of these datasets is an inherent limitation. Second, as the field evolves, more disulfidptosis-related genes will likely be discovered, and their incorporation could refine our model; furthermore, comparative benchmarking is challenged by the proliferation of diverse models with varying biological bases, data sources, and analytical methods, making direct performance comparisons difficult. Third, regarding clinical application, the predictive value of DisulfidpScore for immunotherapy response requires validation in prospective cohorts. Finally, the potential therapeutic value and underlying mechanisms of the candidate drugs identified (e.g., elesclomol) need to be rigorously explored through future *in vitro*, *in vivo*, and clinical studies to confirm their efficacy and safety.

## Resource availability

### Lead contact

Requests for further information and resources should be directed to and will be fulfilled by the lead contact, Quhuan Li (liqh@scut.edu.cn).

### Materials availability

The present study did not generate new unique reagents.

### Data and code availability

• This paper analyzes existing, publicly available data, accessible at TCGA: TCGA-Gliomas; GTEx: Normal brain; CGGA: CGGA325, CGGA693; GlioVis: GSE16011, GSE108474, GSE4271, GSE4412, and E_MATE_3892; and TIGER: GBM-PRJNA482620.
• The code supporting the findings of this study is available in GitHub at https://github.com/clarozhong/Rdata.git.
• Any additional information required to reanalyze the data reported in this paper is available from the lead contact upon request.

## Acknowledgments

This work was funded by the 10.13039/501100001809National Natural Science Foundation of China (grant numbers 31870928 and 32271360), the 10.13039/501100003453Natural Science Foundation of Guangdong Province, China (grant numbers 2021A1515010040 and 2023A1515010829), and Ganzhou Science and Technology-Healthcare Joint Program (grant number 2025YLCE0231).

## Author contributions

Conceptualization, R.H. and Q.L.; methodology, R.H. and Q.L.; investigation, data curation, and formal analysis, R.H., H.L., Y.Z., P.Z., Y.W., A.Y., Y.Z., and J.L.; writing – original draft, R.H.; writing – review & editing, Q.L. and R.X. All authors have read and approved the final manuscript.

## Declaration of interests

The authors declare no competing interests.

## STAR★Methods

### Key resources table

**Table:** 

REAGENT or RESOURCE	SOURCE	IDENTIFIER
**Deposited data**		

TCGA	https://xenabrowser.net/datapages/	TCGA: TCGA-Gliomas
GTEx	https://www.gtexportal.org/home/	GTEx: Normal brain
CGGA	https://www.cgga.org.cn/	CGGA: CGGA325, CGGA693
GLIOVIS	http://gliovis.bioinfo.cnio.es	GLIOVIS: GSE16011, GSE108474, GSE4271, GSE4412 and E_MATE_3892
TIGER	http://tiger.canceromics.org/	TIGER: GBM-PRJNA482620

**Software and algorithms**		

R (v 4.3.3)	The R Project	https://cran.rstudio.com
Code	This paper	https://github.com/clarozhong/Rdata.git
maftools	Mayakonda et al.^38^	https://bioconductor.org/packages/release/bioc/html/maftools.html
CIBERSORT	Chen et al.^39^	https://cibersort.stanford.edu/
IOBR	Zeng et al.^40^	https://github.com/IOBR/IOBR
ImmuneSubtypeClassifier	Thorsson et al.^41^	https://github.com/irjc/ImmuneSubtypeClassifier
pRRophetic	Geeleher et al.^42^	https://github.com/paulgeeleher/pRRophetic
scTenifoldKnk	Osorio et al.^43^	https://github.com/cailab-tamu/scTenifoldKnk

### Experimental model and study participant detail

Using “Glioma” as the search term, we systematically queried the public repositories TCGA, GTEx, CGGA and GLIOVIS up to June 30, 2024. Only ‘‘Homo sapiens’’ datasets explicitly annotated as Gliomas were retained. Selection criteria are: (i) sample size >50; (ii) availability of prognostic information. Consequently, the following datasets were adopted for model construction and validation: TCGA: TCGA-Gliomas; GTEx: Normal brain; CGGA: CGGA325, CGGA693; and GLIOVIS: GSE16011, GSE108474, GSE4271, GSE4412, E_MATE_3892.

### Method details

#### Data collection and preprocessing

RNA-seq gene expression files, clinical information, and genomic mutation data (including single nucleotide variants [SNV] and copy number variations [CNV]) for LGG and GBM were obtained from TCGA and downloaded using the UCSC Xena platform. Normal brain sample RNA-Seq gene expression datasets were retrieved from the GTEx database. For the CGGA325 and CGGA693 cohorts, RNA-Seq gene expression data and related clinical data were retrieved from the CGGA database. Gene expression matrices and clinical information for five glioma cohorts (GSE16011, GSE108474, GSE4271, GSE4412, and E-MATE-3892) were retrieved from the GLIOVIS database. Clinical data and RNA-Seq gene expression files for the GBM-PRJNA482620 immunotherapy cohort were sourced from the TIGER database (Table 1). The RNA-seq data were converted to TPM format and log2-transformed. The microarray data underwent normalization and background correction using the Affy package. A total of eight glioma cohorts, specifically TCGA-gliomas, CGGA325, CGGA693, GSE16011, GSE108474, GSE4271, GSE4412, and E-MTAB-3892, were integrated into a unified cohort for comprehensive analysis. Batch effects across cohorts were adjusted using the ComBat function from the sva package, with parametric empirical Bayes priors enabled (par.prior = TRUE) and both location and scale parameters estimated (mean.only = FALSE). Cohort identity served as the batch variable. The efficacy of batch correction was assessed through PCA visualization. Subsequently, the LGG and GBM cohorts were extracted from the integrated meta-cohort for further analysis. Fifteen disulfidptosis genes, including *ACTB, ACTN4, CAPZB, CD2AP, DSTN, FLNA, FLNB, INF2, IQGAP1, MYL6, MYH9, MYH10, PDLIM1, SLC7A11,* and *TLN1*, were identified in a recent study.^9^

**Table 1:** An overview of the dataset

Accession/Cohort	Database	RNA library	Sample size
TCGA-Gliomas	TCGA	RNA sequencing	LGG:528, GBM:168
GTEx	GTEx	RNA sequencing	normal brain:289
CGGA325	CGGA	RNA sequencing	LGG:182, GBM:139
CGGA693	CGGA	RNA sequencing	LGG:443, GBM:249
GSE16011	GEO	microarray	LGG:117, GBM:159
GSE108474	GEO	microarray	LGG:225, GBM:219
GSE4271	GEO	microarray	LGG:24, GBM:76
GSE4412	GEO	microarray	LGG:26, GBM:59
E_MATE_3892	ArrayExpress	microarray	LGG:154, GBM:16
GBM-PRJNA482620	BioProject	RNA sequencing	responder:17; non-responder:17

#### GSCA analysis

The GSCA database (https://guolab.wchscu.cn/GSCA/#/) was utilized to explore the relationship between disulfidptosis genes and SNV, CNV, DNA methylation, and expression levels across the pan-cancer data.

#### Genomic feature analysis

The maftools package was used to analyze the somatic mutation data to investigate the mutation frequency and types of specific genes in different groups, examine the relationships between gene mutations, compare gene mutation differences between groups, and calculate the tumor mutation burden and mutation count in the samples. Copy number variation data were analyzed using GISTIC2.0 to obtain information regarding gene amplification and deletion. The ggplot2 package was used to visualize the frequency, types, and chromosomal distribution of CNVs in specific genes.

#### Unsupervised clustering analysis

To identify distinct disulfidptosis patterns in gliomas, we conducted univariate Cox regression analysis to screen for disulfidptosis-related genes that are significantly associated with the prognosis of glioma patients. The selection criterion was set at *p* < 0.05 in at least five glioma cohorts. Unsupervised clustering was conducted using the k-means algorithm in the ConsensusClusterPlus package upon the expression levels of prognostic disulfidptosis genes across eight cohorts. This process included 1,000 iterations to ensure the stability of the clustering results, with the following specifications: cluster numbers ranging from 2 to 6 (maxK = 6), 1,000 resampling iterations (reps = 1000), 80% sample subsampling (pItem = 0.8), complete feature retention (pFeature = 1), k-means algorithm (clusterAlg = “km”), Euclidean distance metric, complete linkage for both inner and final hierarchical clustering, and a fixed random seed (seed = 1234) to ensure reproducibility. The optimal cluster number was determined by the Proportion of Ambiguous Clustering (PAC) metric, defined as the area under the cumulative distribution function of consensus matrix values between 0.1 and 0.9 (PAC = F[0.9] - F[0.1]); the solution yielding the minimal PAC value was selected. The sample clustering results were validated using Principal Component Analysis (PCA), and the distribution of disulfidptosis genes and clinical characteristics across different disulfidptosis patterns were visualized using the pheatmap package. Survival analysis was performed to compare survival differences among different disulfidptosis patterns.

#### Immune microenvironment analysis

To comprehensively evaluate the abundance of immune cell infiltration in patients with gliomas, multiple immune infiltration analysis algorithms were employed, including CIBERSORT, QuantiSeq, MCP-counter, xCELL, EPIC, and IPS. Quantification of the proportion of infiltrating immune cells was done using CIBERSORT software. The ESTIMATE algorithm was employed to calculate the immune, stromal, and ESTIMATE scores for glioma patients. The IOBR package was used to download the cancer immune cycle, immune function, and TIME-related gene sets along with the Tumor Immune MicroEnvironment (TIME) characteristic gene sets reported by Kobayashi et al.^44^ and Bagaev et al.^45^ The enrichment of these features was assessed using the ssGSEA algorithm. Between-group comparisons of immune scores were conducted using the Wilcoxon rank-sum test, with Benjamini-Hochberg false discovery rate adjustment applied to account for multiple testing. Immune subtypes were identified using the ImmuneSubtypeClassifier software package.

#### Pathway enrichment analysis

To explore pathway differences between various disulfidptosis patterns, Hallmark gene sets were downloaded from MSigDB database for Gene Set Variation Analysis (GSVA) analysis. The limma package was utilized for differential analysis, with pathways considered significant at *p* < 0.05. Specifically, differential pathway activity was assessed using empirical Bayes-moderated t-statistics, followed by Benjamini-Hochberg false discovery rate correction. To further compare disulfidptosis pattern-related pathways at the transcriptional level, gene sets from ten classical cancer signaling pathways were collected.^46^ The ssGSEA algorithm was then used to compute enrichment scores. Additionally, we performed differential analysis of samples from different disulfidptosis patterns, identifying genes that have p.adjust <0.01 and |log2FC| > 0.5 as differentially expressed genes (DEGs). The clusterProfiler package was employed for GSEA of the identified DEGs based on c2.cp.kegg.v2023.2. The Hs. symbols represent gene sets used to identify specific pathways of different disulfidptosis patterns. Pathways with FDR<0.25 and p.adjust<0.05 were deemed to be statistically significant.

#### Construction and validation of the prognostic model

To enhance comparability across different cohorts, gene expression data from all cohorts were transformed into Z-scores. The prognostic model was constructed and validated using eight glioma cohorts with survival information, including TCGA-Gliomas, CGGA325, CGGA693, GSE16011, GSE108474, GSE4271, GSE4412, and E-MATE-3892. The model construction process consisted of the following steps:

Step 1: Differential Analysis. A differential analysis was performed on each of the eight glioma cohorts using the limma package, with DEGs defined as |log2FC|≥0.5 and p.adjust<0.01. Glioma-specific disulfidptosis-related genes were defined as those that were consistently upregulated or downregulated in at least seven cohorts. The 53 DEGs with prognostic significance were found using univariate Cox regression analysis. These 53 prognostic DEGs and 9 disulfidptosis prognostic genes were used for constructing a prognostic model.

Step 2: Model Construction Using Machine Learning. Ten algorithms were evaluated through 101 combinations. Implementation specifications: (1) Random Survival Forest: 100 trees, node size 3, log rank splitting; (2) Elastic Net: 10-fold CV, C-index metric, 11 alpha values (0–1); (3) Stepwise Cox: bidirectional selection, AIC criterion; (4) CoxBoost: 100 iterations, penalty 9; (5) PlsRcox: 10 components, 10-fold CV; (6) SuperPC: 20 thresholds, 3 components; (7) GBM: 1,000 trees, depth 3, learning rate 0.01; (8) Survival-SVM: van Belle kernel, gamma 0.1; (9) Ridge: L2 penalty, 10-fold CV; (10) LASSO: L1 penalty, 10-fold CV. The TCGA cohort served as the training set (*n* = 691), with the remaining seven cohorts constituting independent validation sets. To mitigate overfitting, each model underwent 10-fold cross-validation repeated across 100 bootstrap resamplings within the training set. Performance was quantified by Harrell’s concordance index. Final model selection prioritized the highest mean C-index averaged across all eight cohorts; the Elastic Net with alpha = 0.3 emerged as the optimal configuration and was designated DisulfidpScore.

Step 3: Model evaluation. Based on the median DisulfidpScore for each cohort, glioma patients were divided into high and low DisulfidpScore groups. Survival and multivariate Cox regression analyses were used to assess the prognostic value of the model. Receiver operating characteristic (ROC) curves and C-index were used to assess the predictive accuracy of the model. Additionally, the constructed model was compared to 50 published glioma-related models to evaluate its superiority.

#### Immunotherapy prediction analysis

The TIDE tool has been used to predict how glioma patients would react to immune checkpoint inhibitor therapy; higher immunotherapy efficacy is correlated with lower TIDE scores.^32^ TIDE scores were computed following *Z* score normalization of expression profiles. Statistical comparisons between DisulfidpScore risk groups employed the Wilcoxon rank-sum test with Benjamini-Hochberg false discovery rate correction for multiple comparisons. A GBM cohort treated with anti-PD-1 therapy was used to validate the predictive ability of the model for immunotherapy. We further performed analyses on key immune checkpoint gene expression, their prognostic impact, correlation with DisulfidpScore, and T cell functional states to investigate mechanisms of immunotherapy resistance.

#### Drug sensitivity analysis

With the pRRophetic package, the half-maximal inhibitory concentration (IC50) of 251 drugs against gliomas was estimated. After normal transformation, the sensitivity to each drug in the different DisulfidPS core groups was visualized. A lower IC50 value indicates that a drug or compound can achieve an inhibitory effect at a lower concentration.^47^ Drugs with significantly different IC50 values between the different DisulfidpScore groups (*p* < 0.01) were screened. Using Pearson correlation analysis, drugs that had a significant association with the Disulfidpscore (|cor|>0.5, *p* < 0.01) were identified. Significance testing employed Student’s *t* test with Benjamini-Hochberg false discovery rate adjustment. The intersection of these results yielded 95 drugs related to the glioma DisulfidpScore, from which 10 drugs with the smallest IC50 values were selected as candidate therapeutic drugs. To predict the clinical performance and potential adverse effects of the candidate therapeutic drugs, the ADMET database (https://admetmesh.scbdd.com/) was used to assess the related pharmacokinetics and safety *in vivo*, including absorption, distribution, metabolism, excretion, and toxicity.

#### Single-cell RNA-seq and in silico perturbation analysis

To investigate the cellular localization and dynamic regulation of disulfidptosis, we integrated single-cell RNA-seq (scRNA-seq) analysis. scRNA-seq datasets for gliomas were obtained from the GEO database. Data processing, including quality control, normalization, and dimensionality reduction, was performed using the Seurat package. Quality control retained cells expressing 200–6,000 genes with mitochondrial content below 20%. Doublet removal was performed using DoubletFinder. Normalization employed log-transformation with scaling factor of 10,000; variable feature selection identified 2,000 top genes by variance-stabilizing transformation. Cell cycle scoring utilized canonical S and G2/M phase gene sets. Batch correction across samples was implemented via Harmony. Cell types were annotated based on canonical markers to identify the specific cell subpopulations harboring disulfidptosis features. The AUCell algorithm was employed to quantify disulfidptosis activity in individual cells using the 15-gene signature. Disulfidptosis heterogeneity was evaluated across different glioma grades (GBM vs. LGG) and cell types. Furthermore, to mechanistically predict the regulatory impact of core disulfidptosis genes, we performed in silico knockout analysis using the scTenifoldKnk package. By comparing the gene regulatory networks (GRNs) between the wild-type and virtual knockout models, we identified downstream targets and pathways dependent on disulfidptosis regulators, providing a mechanistic basis for the subsequent bulk-level analysis.

### Quantification and statistical analysis

R version 4.3.3 and the corresponding packages were used for all statistical analyses. The Wilcoxon rank-sum test was used for data that was not regularly distributed between two groups, while the Student’s *t* test was used for data that was normally distributed for continuous variables. For data that was regularly distributed, a one-way ANOVA was employed; for data that was not normally distributed among three or more groups, the Kruskal-Wallis test was employed. The chi-squared test was used to analyze categorical variables. Multiple comparisons were adjusted via Benjamini-Hochberg false discovery rate control unless otherwise indicated. Machine learning validation reports mean concordance indices with standard deviations across bootstrap iterations; model comparisons utilized paired t-tests. The survival package was used for univariate and multivariate Cox regression analyses and Kaplan-Meier survival analysis, with log rank tests used to determine survival differences. The ROC curves were plotted using the TimeROC package. The chromosomal distribution of the disulfidptosis genes was shown using the RCircos package. The associations between the variables were investigated using Pearson’s correlation analysis. Statistical symbolic significance: ns (not statistically significant.) *p* > 0.05; ∗*p* < 0.05; ∗∗*p* < 0.01; ∗∗∗*p* < 0.001; and ∗∗∗∗*p* < 0.0001.
