# Supplementary material for: Integration of machine learning to develop a disulfidptosis model for predicting glioma prognosis, immunotherapy response, and drug
Source: iScience. 2026 Apr 8;29(5):115657. doi: 10.1016/j.isci.2026.115657 (PMC13185775; doi:10.1016/j.isci.2026.115657)
Supplement: Document S1. Figures S1–S8 [file mmc1.pdf]

iScience, Volume *29*

Supplemental information

Integration of machine learning to develop a disulfidptosis model for predicting glioma prognosis, immunotherapy response, and drug

Ruiting Huang, Hailin Li, Yijing Zhong, Paimin Zhuo, Yibei Wang, Aoting Yang, Yu Zhang, Jiao Li, Ruiquan Xu, and Quhuan Li

Supplementary Materials

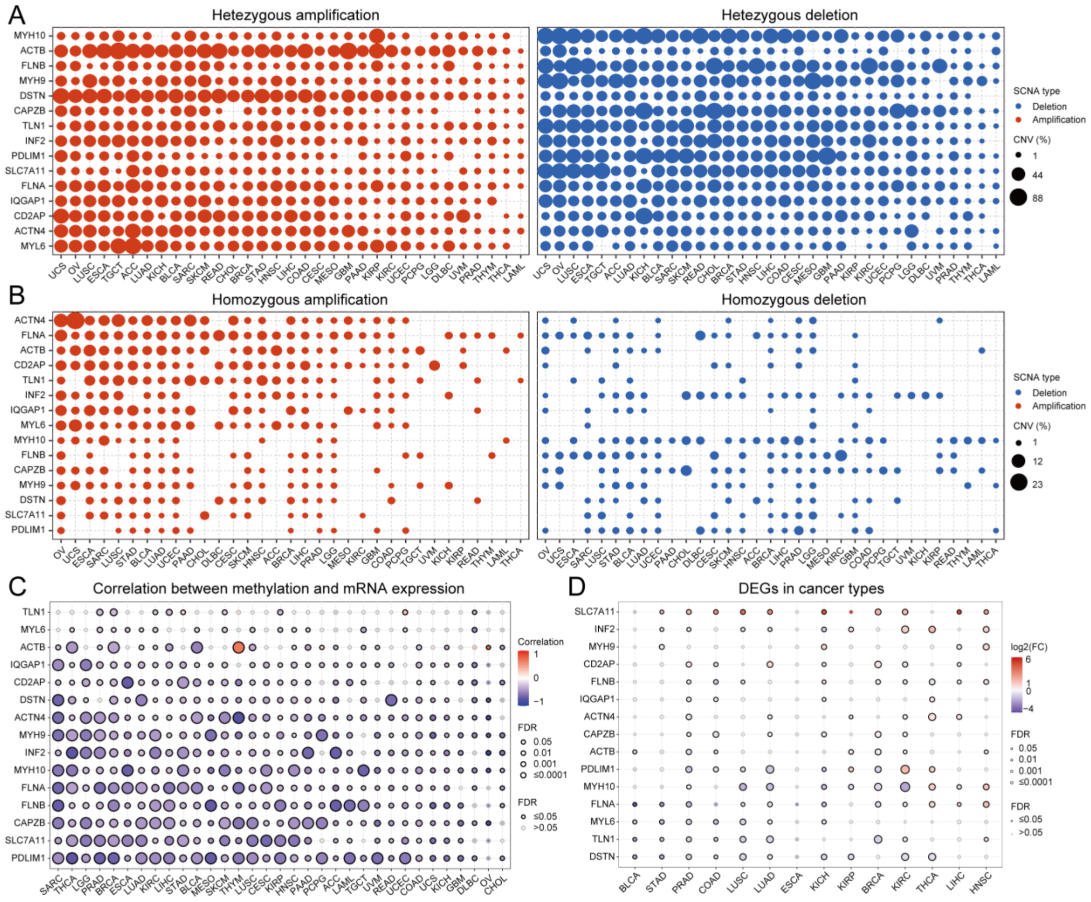

Fig. S1 Transcriptomic and Epigenetic Landscape of Disulfidptosis Genes in Pan-Cancer. (A-B) Distribution of heterozygous and homozygous CNV of disulfidptosis genes in pan-cancer. (C) Correlation between the expression of disulfidptosis genes and their DNA methylation in pan-cancer. (D) Expression differences of disulfidptosis genes across pan-cancer

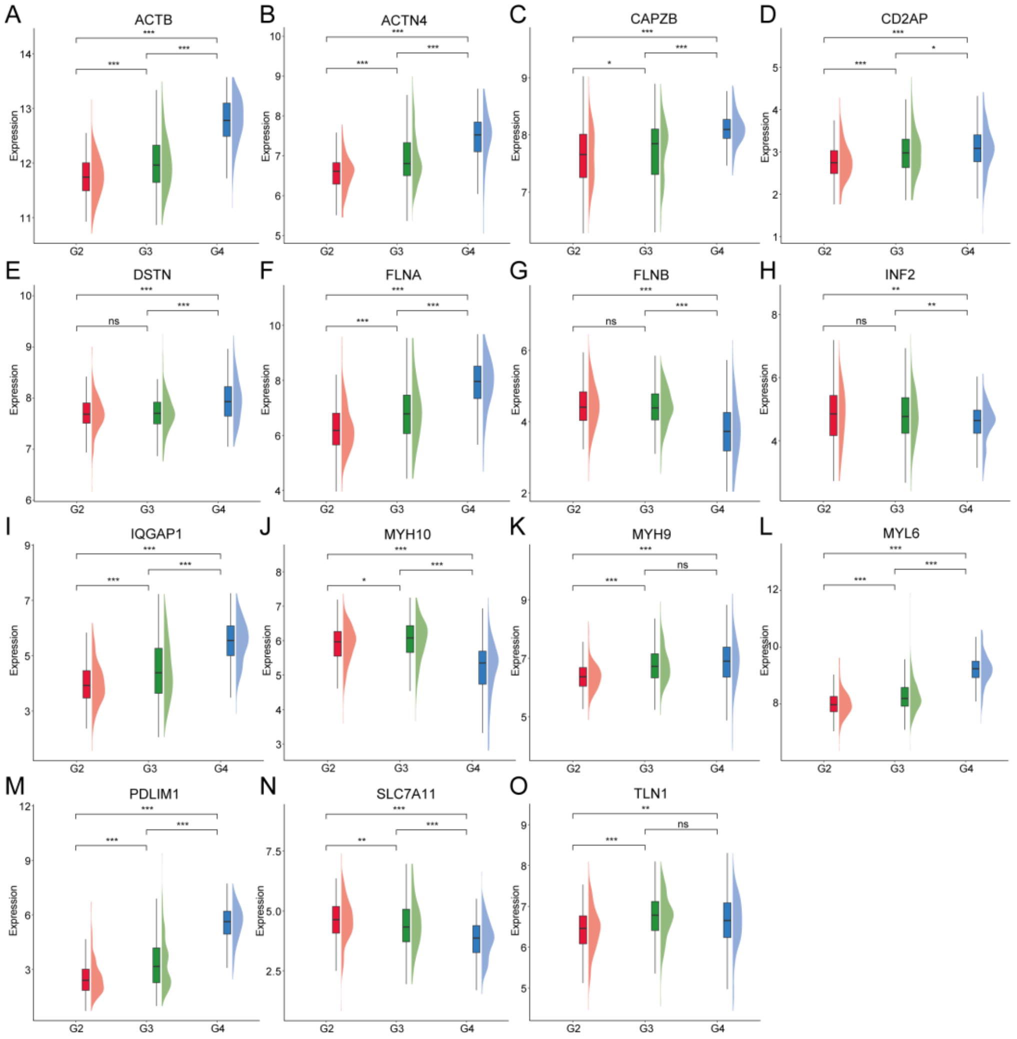

Fig. S2 Expression Differences of Disulfidptosis Genes in Different Pathological Grades of Gliomas. ***p < 0.001, **p < 0.01, *p < 0.05, ns: not statistically significant

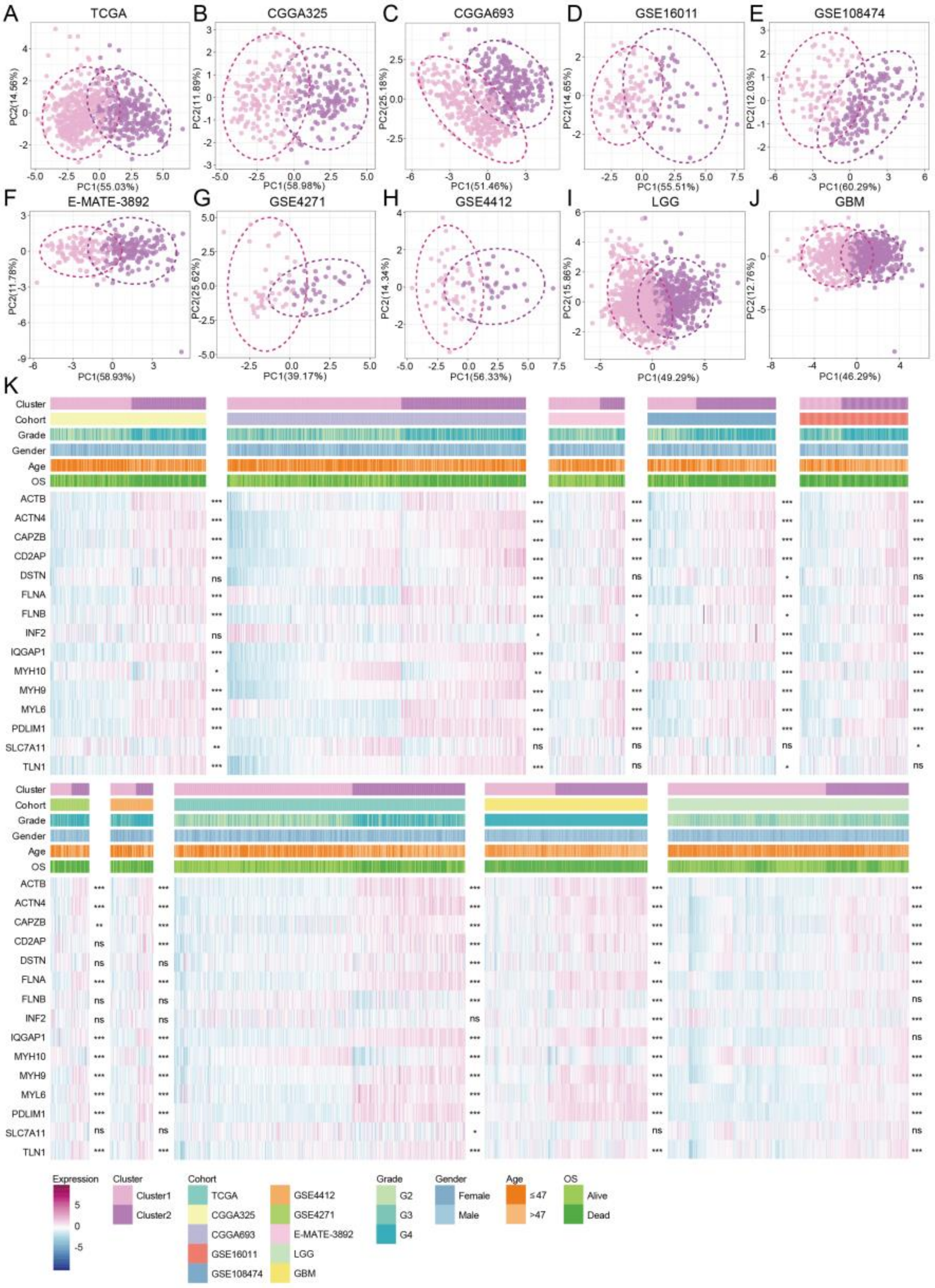

Fig. S3 Sample Distribution, Molecular Expression, and Clinical Characteristics Comparison of Disulfidptosis Patterns in Different Glioma Cohorts. (A-J) Sample distribution of two disulfidptosis patterns in TCGA, CGGA325, CGGA693, GSE16011, GSE108474, E-MTAB-3892, GSE4271, GSE4412, LGG, and GBM cohorts. (K) Heatmap showing the expression levels of disulfidptosis genes and distribution of clinical characteristics under two disulfidptosis patterns across 10 cohorts. ***p<0.001, **p<0.01, *p<0.05, ns: not statistically significant

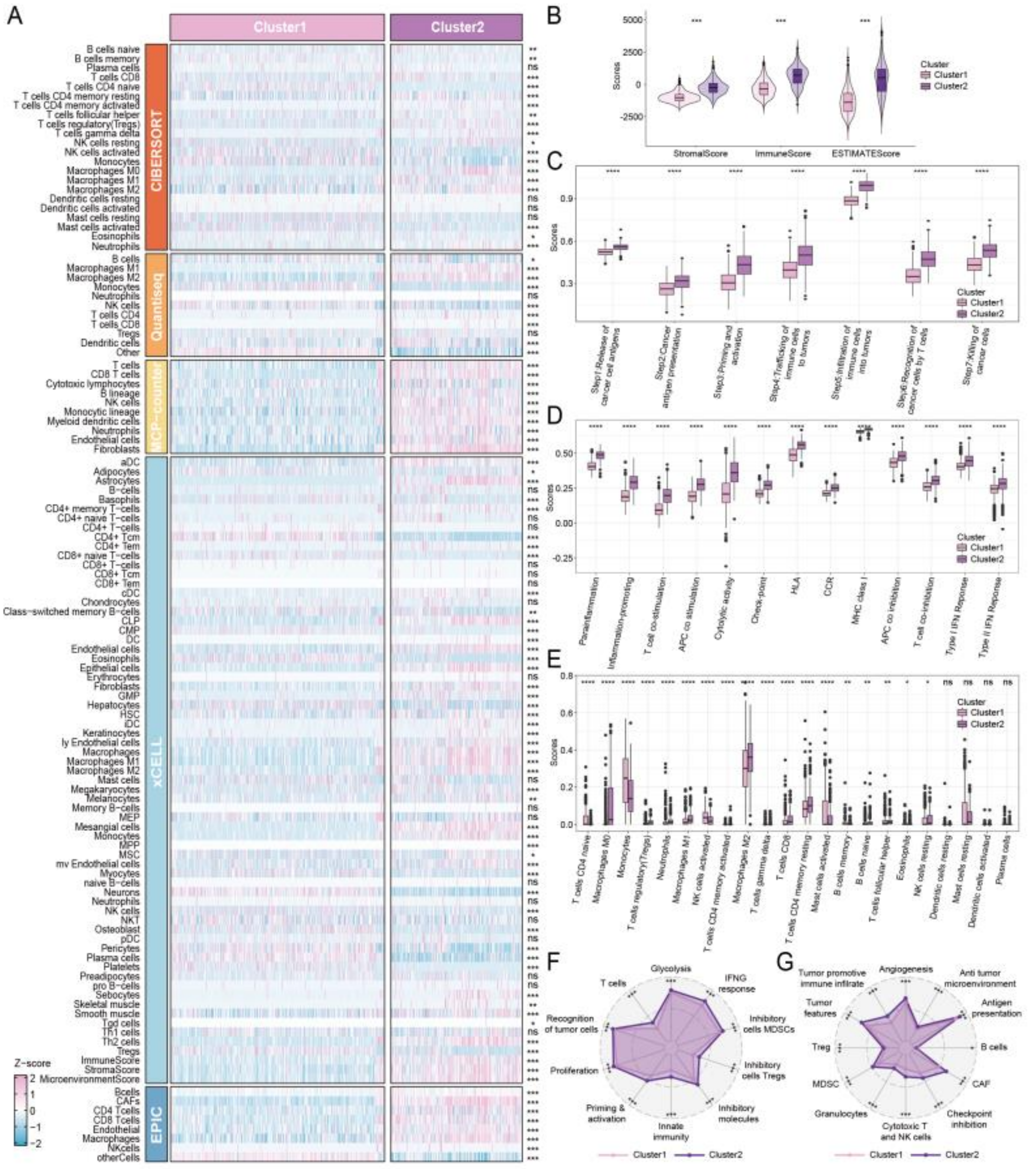

Fig. S4 Immune Infiltration Differences in Gliomas with Different Disulfidptosis Patterns. (A) Immune cell infiltration distribution between different disulfidptosis patterns analyzed by six algorithms. (B) Differences in stromal scores, immune scores, and ESTIMATE scores between different disulfidptosis patterns in gliomas. (C-E) Differences in the cancer immunity cycle, immune functions, and immune cell infiltration between different disulfidptosis patterns in gliomas. (F-G) Differences in TIME features developed by Kobayashi and Bagaev between the two disulfidptosis patterns in gliomas. ****$p<0.0001$, ***$p<0.001$, **$p<0.01$, *$p<0.05$, ns: not statistically significant

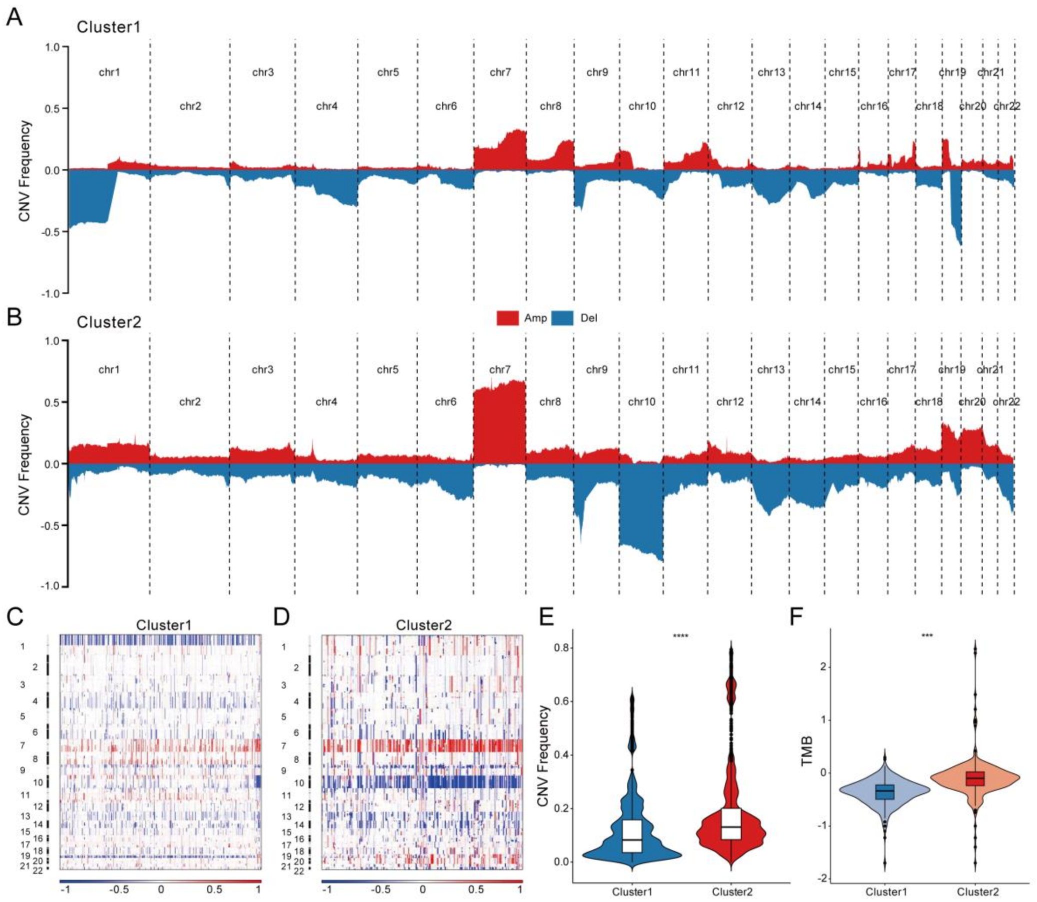

Fig. S5 Genomic Characteristic Differences in Gliomas with Different Disulfidptosis Patterns. (A-B) Chromosomal distribution of CNV frequency in Cluster 1 and Cluster 2 in gliomas with different disulfidptosis patterns. (C-D) The heatmap illustrates the chromosomal alterations in Cluster 1 and Cluster 2 in gliomas with different disulfidptosis patterns. (E) Differences in CNV frequency between different disulfidptosis patterns in gliomas. (F) Differences in TMB between different disulfidptosis patterns in gliomas. ****p < 0.0001, ***p < 0.001. TMB: Tumor Mutation Burden

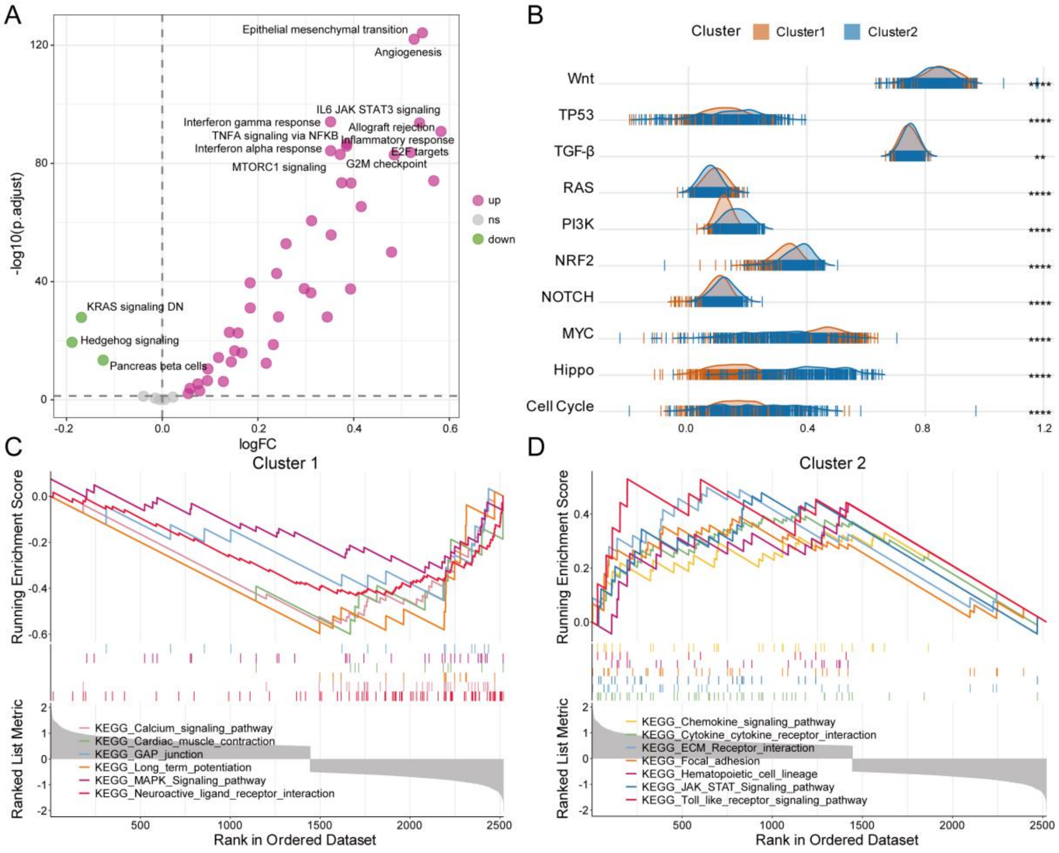

Fig. S6 Signaling Pathway Differences in Gliomas with Different Disulfidptosis Patterns. (A) Differences in Hallmark pathway activities between different disulfidptosis patterns in gliomas. Pink labels indicate significant activation of the pathway in Cluster 2, green labels indicate significant activation in Cluster 1, and gray labels indicate no significant difference in pathway activity between the two patterns. (B) Differences in activities of ten classical cancer signaling pathways between different disulfidptosis patterns in gliomas. (C-D) GSEA enrichment pathways in Cluster 1 and Cluster 2 in gliomas with different disulfidptosis patterns. ****$p < 0.0001$, **$p < 0.01$

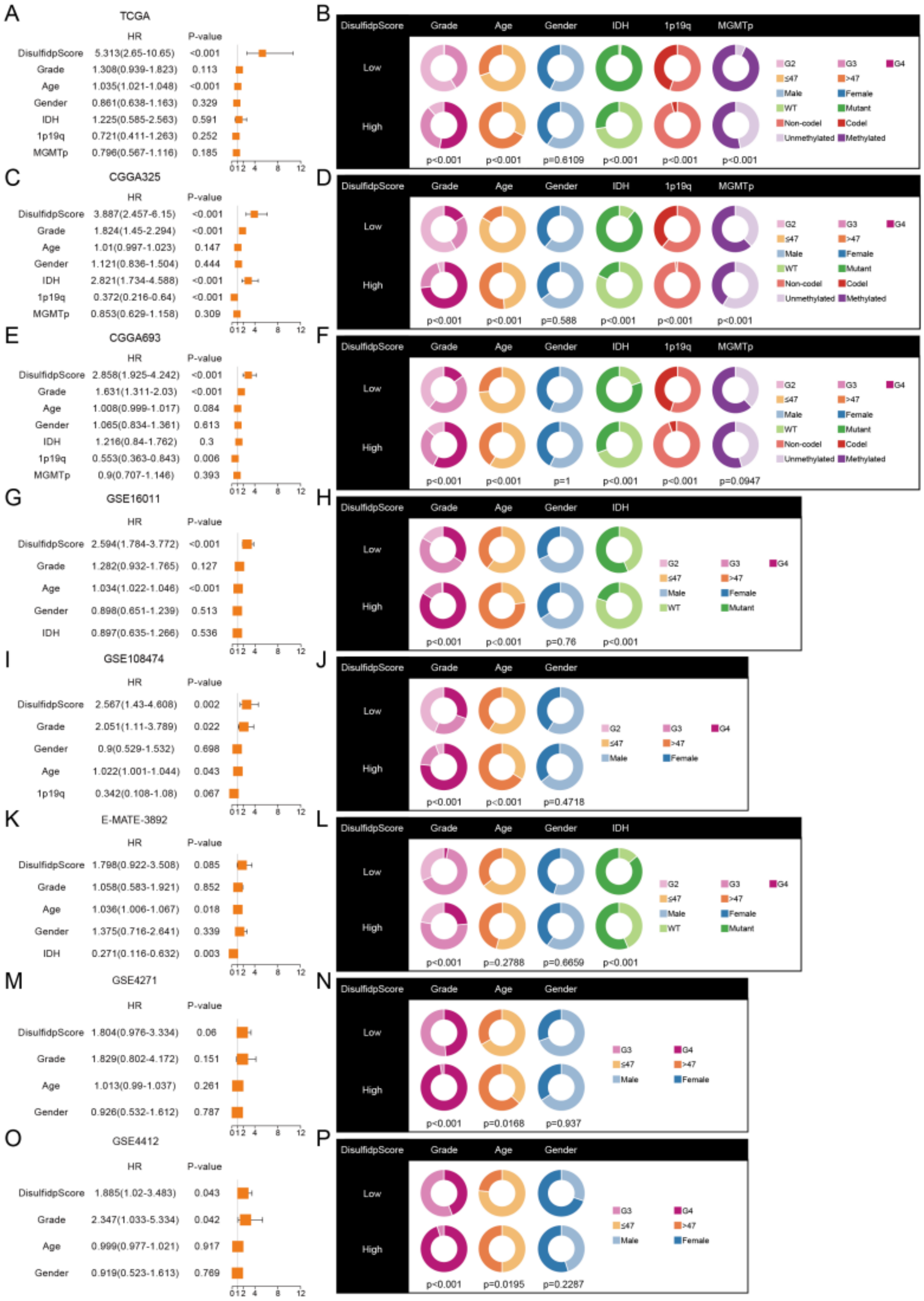

Fig. S7 Independent Prognostic Evaluation of DisulfidpScore in Gliomas and Its Association with Clinical Characteristics. (A-P) Multivariate Cox regression analysis and distribution of clinical characteristics in different DisulfidpScore groups in TCGA (A-B), CGGA325 (C-D), CGGA693 (E-F), GSE16011 (G-H), GSE108474 (I-J), E-MTAB-3892 (K-L), GSE4271 (M-N), and GSE4412 (O-P) cohorts

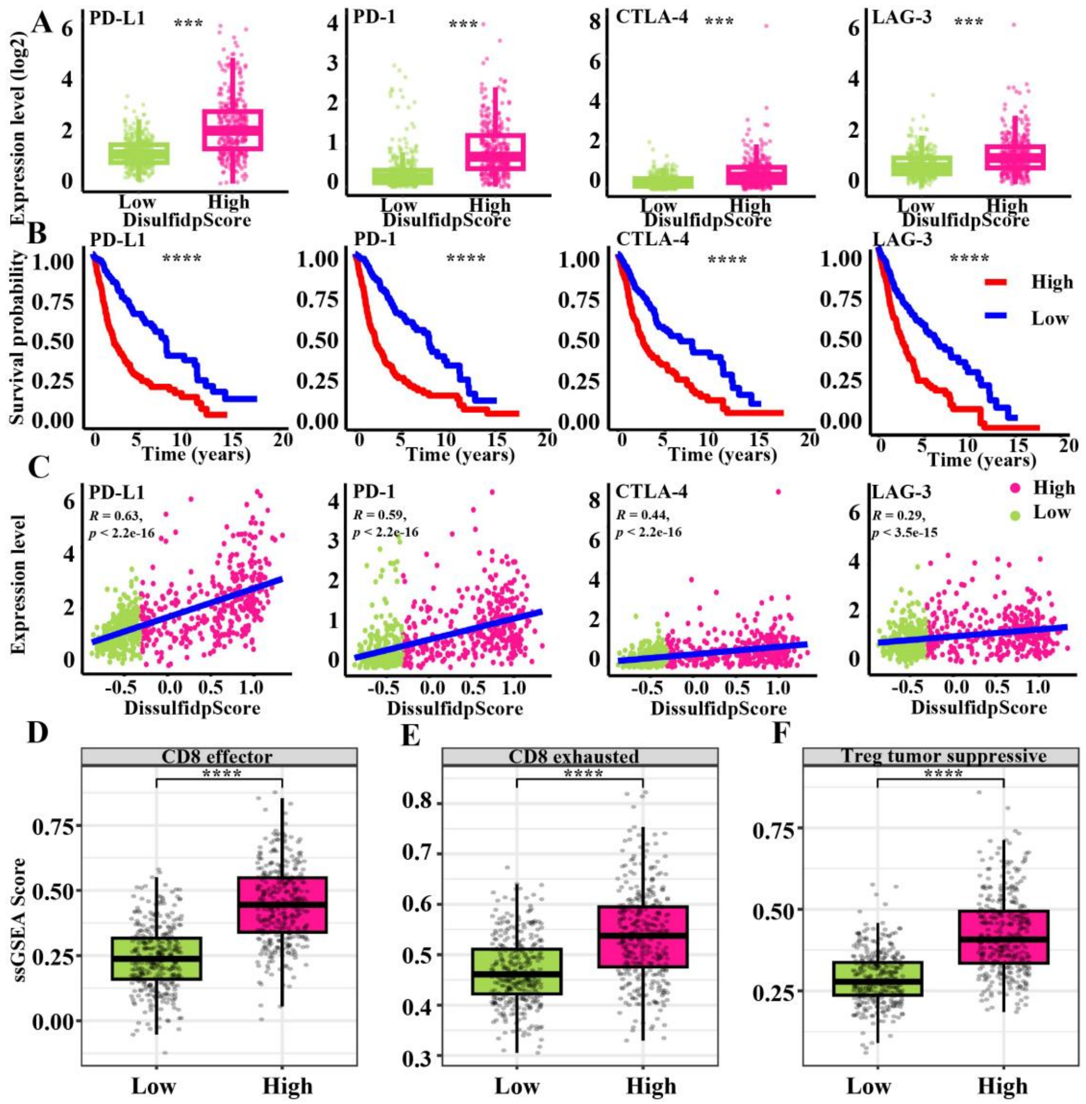

Fig. S8 Analysis of the association between DisulfidpScore and immune checkpoint expression, prognosis, and immune cell infiltration. (A) Box plots showing the expression levels of PD-L1, PD-1, CTLA-4, and LAG-3 in low and high DisulfidpScore groups. (B) Kaplan-Meier survival curves for patients with low and high DisulfidpScore, stratified by the expression of PD-L1, PD-1, CTLA-4, and LAG-3. (C) Scatter plots depicting the correlation between DisulfidpScore and the expression levels of PD-L1, PD-1, CTLA-4, and LAG-3. (D-F) Box plots showing the infiltration levels of CD8 effector T cells, CD8 exhausted T cells, and Treg cells in low and high DisulfidpScore groups. ****$p < 0.0001$, ***$p < 0.001$

.
